# Mannose-decorated cyclodextrin vesicles: The interplay of multivalency and surface density in lectin–carbohydrate recognition

**DOI:** 10.3762/bjoc.8.175

**Published:** 2012-09-17

**Authors:** Ulrike Kauscher, Bart Jan Ravoo

**Affiliations:** 1 Organic Chemistry Institute, Westfälische Wilhelms-Universität Münster, Correnstraße 40, 48149 Münster, Germany

**Keywords:** carbohydrates, cyclodextrins, lectins, molecular recognition, multivalency, vesicles

## Abstract

Cyclodextrin vesicles are versatile models for biological cell membranes since they provide a bilayer membrane that can easily be modified by host–guest interactions with functional guest molecules. In this article, we investigate the multivalent interaction of the lectin concanavalin A (ConA) with cyclodextrin vesicles decorated with mannose–adamantane conjugates with one, two or three adamantane units as well as one or two mannose units. The carbohydrate–lectin interaction in this artificial, self-assembled glycocalyx was monitored in an agglutination assay by the increase of optical density at 400 nm. It was found that there is a close relation between the carbohydrate density at the cyclodextrin vesicle surface and the multivalent interaction with ConA, and the most efficient interaction (i.e., fastest agglutination at lowest concentration) was observed for mannose–adamantane conjugates, in which both the cyclodextrin–adamantane and the lectin–mannose interaction is inherently multivalent.

## Introduction

The surface modification of materials with carbohydrates has attracted much attention due to the fact that such materials can be compared to and compatible with the cell surface [1]. The “glycocalyx” is a dense layer on the surface of the cell, which serves as a responsive interface with its environment and also serves as a natural protective shield. The glycocalyx consists of various numbers and arrangements of polysaccharides and is found in eukaryotic as well as in prokaryotic cells. A well-known example of the pivotal role of oligosaccharides on cell surfaces is the fact that human blood types (A, B, AB and 0) are solely determined by minor changes in the composition of the erythrocyte glycocalyx. Additionally, many biological mechanisms are mediated by multivalent recognition of carbohydrates. For example, lectins are proteins that bind to specific carbohydrates on the cell surface and activate biochemical responses [2]. In this way, protein–carbohydrate interactions regulate cell division, protein synthesis, the immune system, and the adhesion of cells. A well-known lectin is concanavalin A (ConA), which can be readily obtained from jack-beans. It has four identical binding sides and binds α-mannose, α-glucose and their derivatives. Because of the importance of carbohydrates and their multivalent recognition by lectins in physiological processes, they are also considered a promising tool for the development of drug-delivery systems [3].

Synthetic bilayer vesicles are a versatile model for biological cell membranes, and there are a substantial number of reports on synthetic glycolipids that mimic the glycocalyx [4–23]. Multivalent guest interaction with the surface of the vesicles has become a useful system to investigate recognition, adhesion and fusion of biological cell membranes [24–26]. In this context, amphiphilic cyclodextrins are a promising platform due to their ability to form stable bilayer vesicles that can be functionalized by self-assembly [27]. To this end, cyclodextrins are modified with long alkyl chains (“tails”) and short oligo(ethylene glycol) head groups. These macrocyclic amphiphiles form unilamellar bilayer vesicles in aqueous solution upon hydration of a thin film cast by evaporation from organic solution and extrusion through a 0.1 μm polycarbonate membrane [27]. The cavities of each cyclodextrin are available to form inclusion complexes with hydrophobic guest molecules. Adamantane is known to be an excellent guest for β-cyclodextrin cavities (*K*_a_ = (2–3) × 10^4^ M^−1^). We were able to recently demonstrate the interaction of monovalent bifunctional guest molecules, containing a maltose or lactose unit and an adamantane unit, with cyclodextrin vesicles, and their ability to agglutinate with lectins [28]. We also showed that agglutination requires a critical density of carbohydrate ligand on the cyclodextrin vesicle surface [29]. In this work we investigate the influence of multivalent recognition by guest molecules with an increasing number of adamantane and mannose units. It is our hypothesis that more adamantane units in the guest molecule lead to higher affinity for the cyclodextrin vesicles due to multivalent interaction at the vesicle surface. In addition, we increased the number of mannose units in the guest molecule, assuming that a high density of carbohydrate is essential for multivalent lectin binding at the vesicle surface.

## Results and Discussion

**Figure 1:**
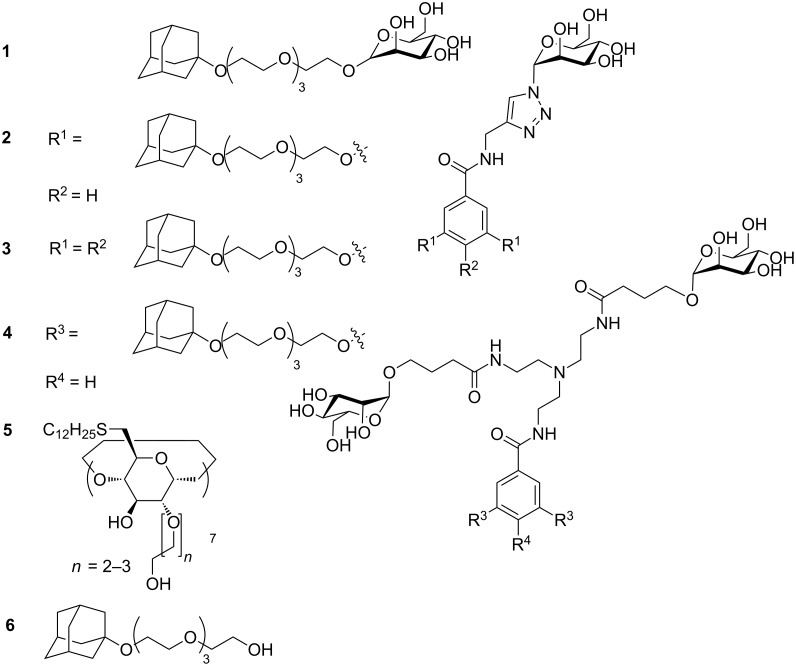
Mannose–adamantane conjugates **1**–**4** and amphiphilic cyclodextrin **5**.

Four different guest molecules were synthesized to study the effect of multivalency, each with a distinct number of adamantane or mannose functions. The adamantane unit can bind into the cavity of cyclodextrins embedded at the vesicle surface. Additionally, all guest molecules possess α-mannose units, which bind to lectins such as concanavalin A (ConA, Figure 1). Guest **1** contains a single mannose and a single adamantane unit. Guest **2** and guest **3** contain a single mannose and two or three adamantane units, respectively. Guest **4** contains two mannose as well as two adamantane units. The synthesis of **1**–**4** is described in Supporting Information File 1. The analytical data for **1**–**4** are fully consistent with their molecular structure. The synthesis of amphiphilic β-cyclodextrin **5** has been reported previously [30]. Unilamellar vesicles with a diameter of 100–150 nm are obtained by extrusion [27,30].

**Figure 2:**
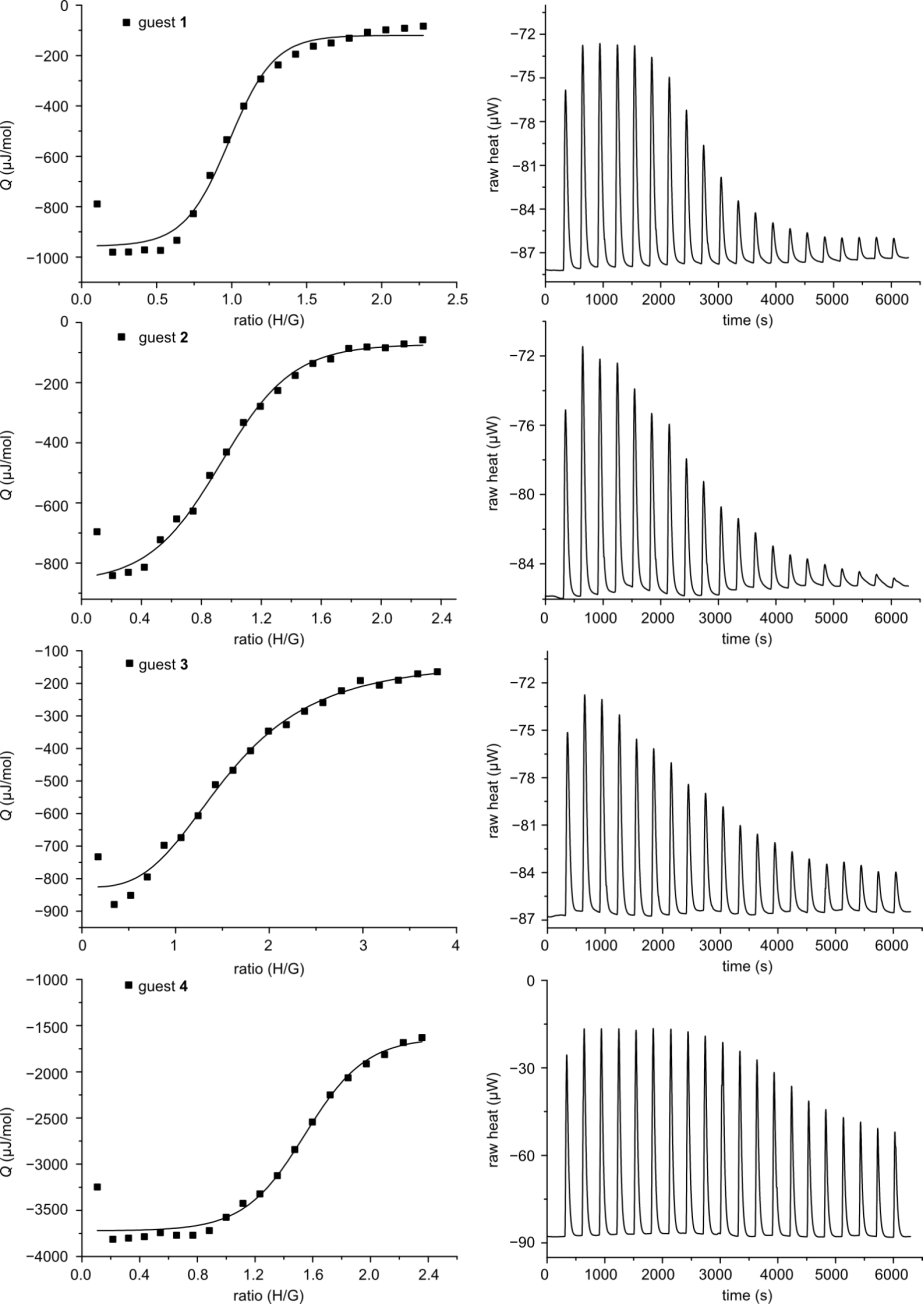
Integrated peak area (left) and raw titration curves (right) for the ITC measurements of **1**–**4** with β-CD. The concentrations used are listed in Table 1.

To investigate the ability of adamantane functions to bind into the cavity of cyclodextrins, the synthesized guest molecules were investigated regarding their 1:1 complexation behavior towards β-cyclodextrin. Isothermal titration calorimetry (ITC) was carried out with β-cyclodextrin and each of the synthesized guest molecules **1**–**4**. The concentrations were chosen to provide one cyclodextrin cavity for each adamantane unit and are displayed in Table 1. The effective adamantane concentration describes the concentration of adamantane units. A guest with two adamantane units (**2** or **4**) results in an effective adamantane concentration that is twice the concentration of the divalent guest molecule. For guest **3**, the effective adamantane concentration is three times the concentration of the trivalent guest molecule. The results of these titrations can be seen in Table 1 and Figure 2.

**Table 1:** Thermodynamic parameters measured with isothermal titration calorimetry.

compound	host	[guest]^a^ mM	[host] mM	Δ*H* kJ/mol	Δ*G* kJ/mol	Δ*S* J/(K·mol)	*K*_a_ M^−1^

**1**	β-CD	5.00	0.41	−18.93	−27.71	29.47	7.2 × 10^4^
**2**^b^	β-CD	0.38	5.0	−16.96	−25.98	30.28	3.6 × 10^4^
**3**^b^	β-CD	0.29	3.6	−28.78	−23.47	−17.82	1.3 × 10^4^
**4**	β-CD	20.0	2.4	−12.62	−24.59	40.19	2.1 × 10^4^

^a^Effective adamantane concentration. ^b^Due to the low solubility of guest molecules the titration was carried out in reverse mode (host added to guest).

**Figure 3:**
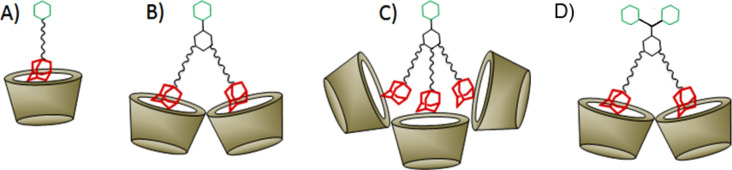
Schematic presentation of the binding between β-cyclodextrin and (A) monovalent guest **1**, (B) divalent guest **2**, (C) trivalent guest **3**, and (D) divalent guest **4**.

The thermodynamic parameters of guests **1**–**4** are characteristic of the formation of a 1:1 inclusion complex of each adamantane unit with β-cyclodextrin. Based on the effective adamantane concentration, the stoichiometry, the binding constants, and the thermodynamic parameters (negative Δ*H* and positive Δ*S*) are very similar for each guest, with the exception of guest **3**. This implies that in guests **1**, **2** and **4**, each and every adamantane unit is able to complex a β-cyclodextrin host molecule independent of the other adamantanes on the guest molecule. A significant deviation of this behavior is observed only for guest **3**, which carries three adamantane units. In this case, the stoichiometry appears to be less than 1:1, the binding constant is somewhat lower, and the thermodynamic parameters are different (notably, Δ*S* is negative). This observation can be explained by steric hindrance in the trivalent host–guest complex: apparently, three β-cyclodextrins are too large to interact efficiently with each of the three adamantane units. The steric bulk of the cyclodextrins hinders the deep intrusion of adamantane functions into the cavity (Figure 3).

The results of the titration calorimetry show that each adamantane on guests **1**–**4** is able to bind to β-cyclodextrin. Accordingly, the guest molecules are expected to form inclusion complexes at the surface of vesicles of amphiphilic cyclodextrin **5**. More importantly, guest molecules **2**–**4** are expected to form multivalent host–guest complexes with a much higher effective binding constant than the monovalent binding constant reported in Table 1. According to a quantitative treatment of multivalent host–guest interactions at surfaces, it may be expected that for a 1:1 monovalent interaction with a binding constant of ≈10^4^ M^−1^, a divalent interaction can have an apparent binding constant of ≈10^7^ M^−1^ and a trivalent interaction can lead to an apparent binding constant of ≈10^10^ M^−1^ [31].

In view of the high affinity binding of the (multivalent) guest molecules **1–4** and assuming that these polar molecules are not able to permeate through the membrane, it is our hypothesis that even at submillimolar concentrations of guest **1–4** and host **5**, most guest molecules are confined to the outer vesicle surface. Moreover, since in all experiments the effective guest concentration is only half of the host concentration, it can be assumed that most cyclodextrin cavities at the vesicle surface are occupied by an adamantane unit. As a consequence, the density of mannose molecules on the surface of the vesicles is expected to be relatively high, resulting in the formation of an artificial glycocalyx by self-assembly. Therefore, the addition of the lectin ConA should lead to agglutination of the vesicles due to the specific interaction of mannose and ConA at the vesicle surface. Optical density measurements were carried out at a wavelength of 400 nm to investigate the agglutination behavior of each guest molecule.

**Figure 4:**
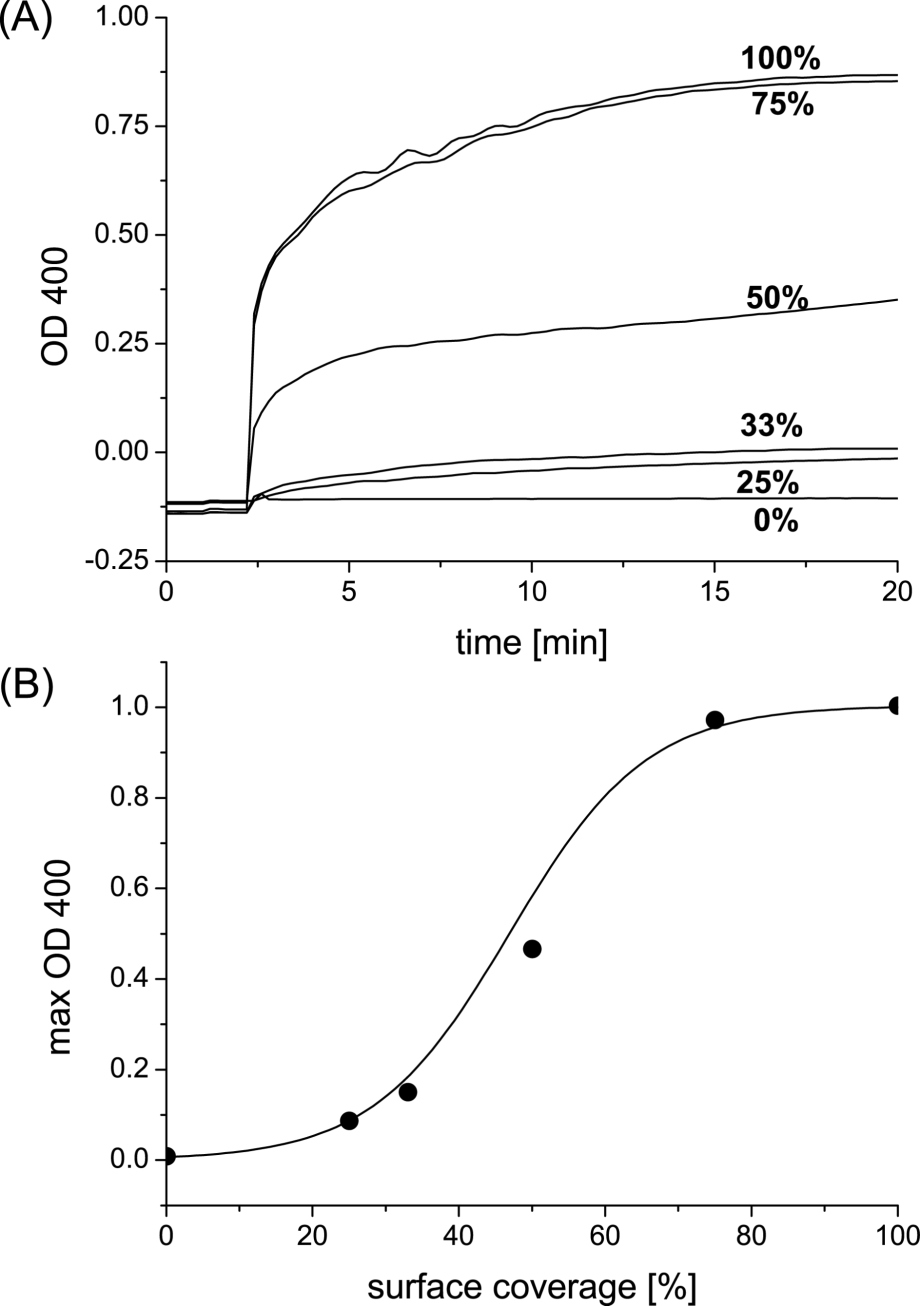
(A) Agglutination of β-cyclodextrin vesicles in the presence of monovalent guest **1** and ConA. The surface coverage of guest **1** is gradually reduced by substitution with “inert” guest **6**. Concentrations: [5] = 0.2 mM, [1] = 0–0.1 mM, [6] = 0–0.1 mM, [ConA] = 0.1 mg/mL. (B) Maximum agglutination depending on surface coverage of guest **1**.

In the case of monovalent guest **1**, a typical time-dependent agglutination was found due to the specific interaction of ConA with mannose at the cyclodextrin vesicle surface (Figure 4). In these experiments, the concentration of cyclodextrin **5** was 0.2 mM and the concentration of guest **1** was 0.1 mM. It was also found that agglutination can be gradually suppressed by the substitution of guest **1** by an “inert” guest (adamantyl tetraethyleneglycol, **6**) that binds cyclodextrin but not ConA (Figure 4). It is evident that as the surface density of mannose is decreased in the glycocalyx, the effective separation of mannose ligands increases, and hence the multivalent interaction of mannose and ConA is suppressed. In fact, a critical threshold is observed around a mannose surface density of 50%: if the mannose density is reduced even further, the average distance of the mannose units on the vesicle surface is larger than the binding site separation of ConA, and hence multivalent interaction is no longer observed. The average distance between two cyclodextrins at the vesicle surface is approximately 2.2 nm [30]. The distance between two mannose molecules is expected to be the same when using a maximum surface coverage of the cyclodextrin host surface with guest **1**. The binding site separation for ConA is 3.6 to 4.9 nm [21–22], which roughly corresponds to the average spacing of mannose at 50% surface coverage of guest **1**. These observations are entirely consistent with our previous investigation of an artificial glycocalyx of lactose and maltose and its interaction with ConA and peanut agglutinin (PNA) [28–29].

**Figure 5:**
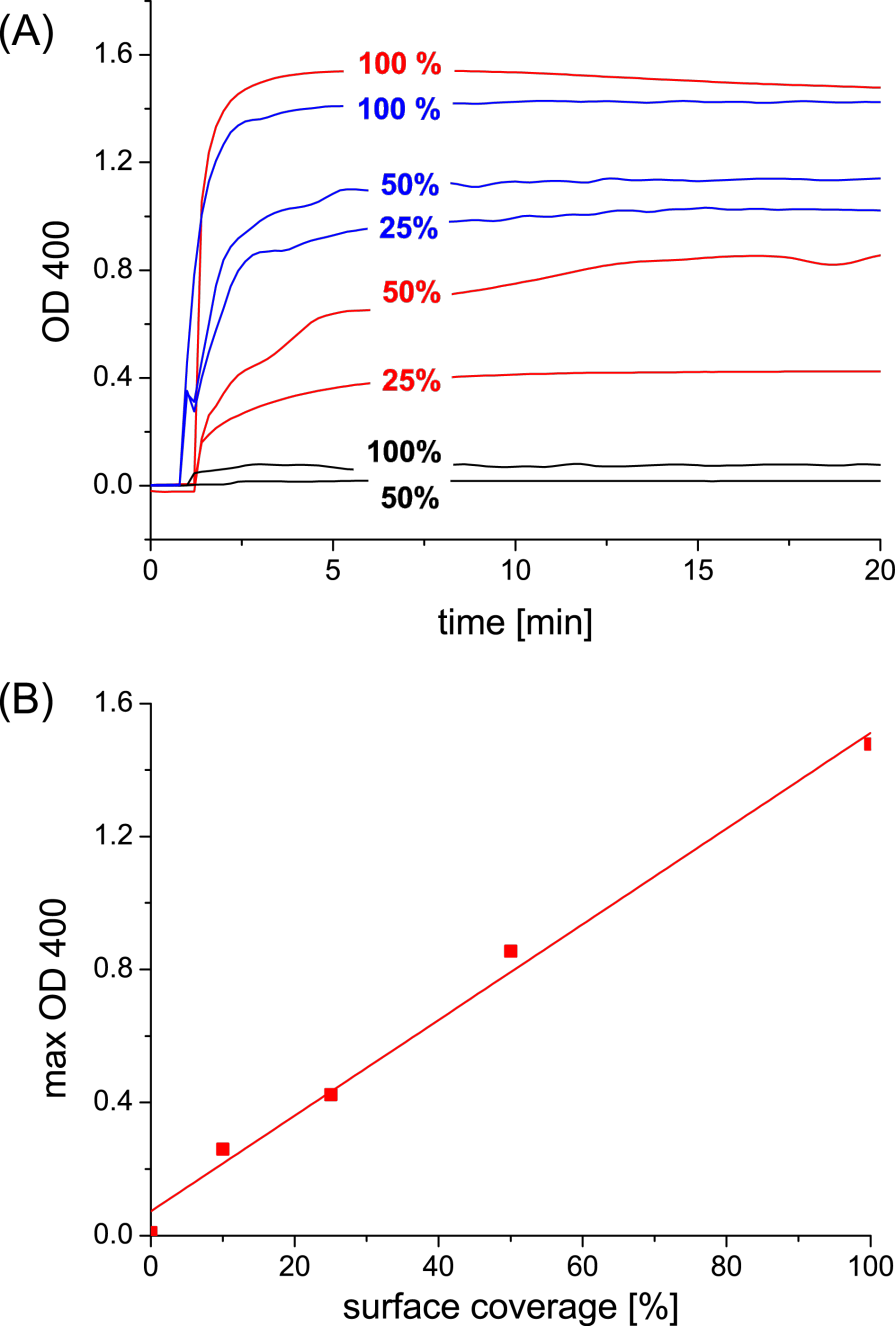
(A) Agglutination dependence of β-cyclodextrin vesicles in the presence of guests **2**–**4**. Legend: red = guest **2**, black = guest **3**, blue = guest **4**. The surface coverage of guests **2**–**4** is gradually reduced by substitution with “inert” guest **6**. Concentrations: [5] = 0.2 mM, [2] = 0–0.05 mM, [3] = 0–0.033 mM, [4] = 0–0.05 mM, [6] = 0–0.075 mM. (B) Maximum agglutination depending on surface coverage of guest **2**.

Interestingly, if guests **2**–**4** (0.1 mM adamantane) were added to cyclodextrin vesicles (0.2 mM) an aggregation effect was detected even in the absence of ConA (Figure 5). This effect is due to noncovalent cross-linking of the vesicles by guest **2**–**4**. Each of the adamantane units on guest **2**–**4** can bind to a different cyclodextrin vesicle (intervesicular binding) and hence cause vesicle aggregation. We have previously observed this effect for homobifunctional guest molecules equipped with two azobenzene, methylbenzoyl, or *tert*-butylbenzyl groups [32–34]. It should be noted that in the case of guest **3** the aggregation due to cross-linking is much smaller, almost invisible, compared to the effect found for guests **2** and **4**. This can possibly be explained by the orientation of the adamantane units in the guest molecules. Guests **2** and **4** have two adamantane units and the average distance between these units is significantly larger than the average distance of the three adamantane units in guest **3**. We propose that due to the adjacency in the case of guest **3**, the binding of one adamantane unit directs the other two units to bind to the same vesicle (intravesicular binding), whereas the larger distance between the adamantane units in guest **2** and **4** allows the adamantane functions to bind to different vesicles (intervesicular binding). As discussed above for guest **1**, the surface density of guests **2**–**4** can be reduced by replacement with “inert” guest **6**. It can be seen from Figure 5 that addition of a substantial amount of this inert competitor effectively reduces the tendency to cross-link the vesicles. As can be seen in the plot of the maximum agglutination induced by guest **2**, a linear dependence (rather than a threshold) between the guest surface coverage and the extent of aggregation is observed. If a higher percentage of cross-linker is present on the vesicle surface, more noncovalent cross-links between vesicles are formed and more extensive aggregation is observed.

**Figure 6:**
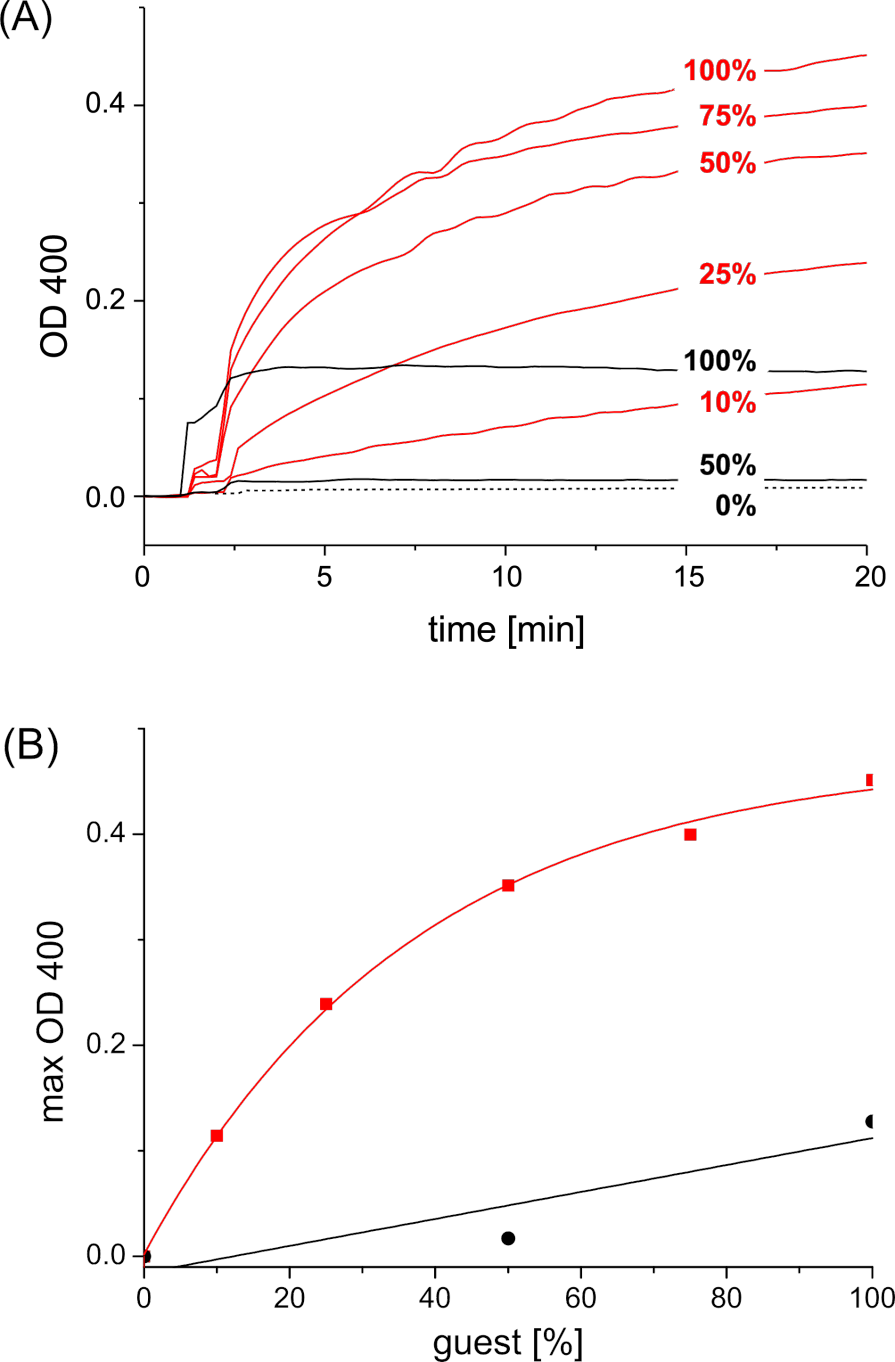
(A) Agglutination of β-cyclodextrin vesicles in the presence of guest **2** or **3** and ConA. Legend: red = guest **2**, black = guest **3**. The surface coverage of guests **2** and **3** is gradually reduced by substitution with “inert” guest **6**. Concentrations: [5] = 20 μM, [2] = 0–5.0 μM, [3] = 0–3.3 μM, [6] = 0–10 μM; [ConA] = 0.1 mg/mL. (B) Maximal aggregation depending on surface coverage of guest **2** or **3**.

In line with the observations discussed above, a tenfold dilution of the concentration of host ([5] = 20 μM) and guest ([2] = [4] = 5.0 μM and [3] = 3.3 μM) results in a complete suppression of cross-linking of the vesicles. Apparently, at this concentration intravesicular binding is favored over intervesicular binding, and vesicle aggregation is negligible. These findings are again consistent with earlier observations [32]. Hence, in this concentration window the agglutination in the presence of ConA can be investigated. Indeed, cyclodextrin vesicles functionalized with guests **2–4** aggregate in the presence of ConA. However, the extent and rate of agglutination is very different for each guest. The observations for divalent guest **2** and trivalent guest **3** are shown in Figure 6.

**Figure 7:**
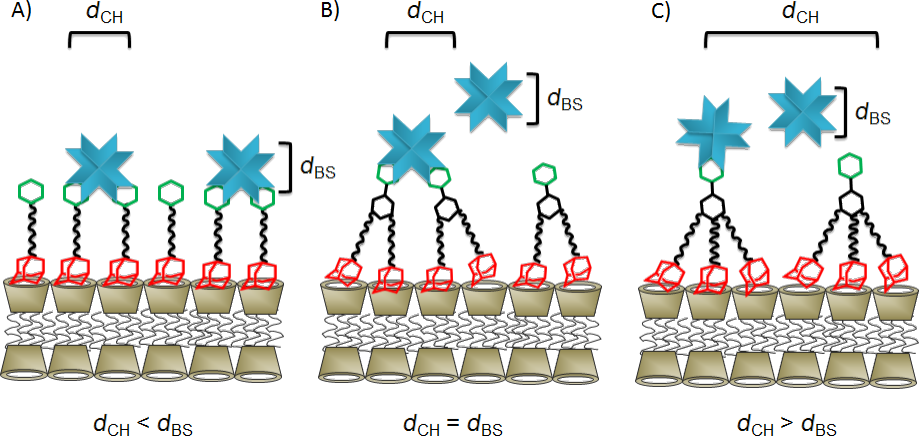
Schematic presentation of the binding of (A) a monovalent, (B) a divalent, or (C) a trivalent guest onto the surface of cyclodextrin vesicles and their interaction with ConA. *d*_CH_: average spacing of carbohydrates, *d*_BS_: effective binding-site separation of ConA.

Perhaps counter intuitively, the extent of agglutination *decreases* with an increasing number of adamantane functions present in the guest molecule. In other words, agglutination is less efficient for **3** compared to **2** in spite of the higher surface affinity of trivalent guest **3** compared to divalent guest **2**. This observation can be explained by the density of mannose on the surface of the vesicles. The average distance between two cyclodextrins at the vesicle surface is approximately 2.2 nm [30]. The distance between two mannose molecules *d*_CH_ is expected to be the same when using a 100% surface coverage of cyclodextrin vesicles with monovalent guest **1**. A decrease in the surface density of mannose due to replacement of guest **1** by “inert” guest **6** decreases the agglutination by ConA (see Figure 4 and Figure 7) since eventually the average spacing of mannose on the vesicle surface (*d*_CH_) exceeds the binding-site separation of ConA (*d*_BS_) [21]. However, the surface density of mannose also decreases if the guest molecule occupies two or even three cyclodextrin cavities but yet carries only one mannose unit (Figure 7).

In the case of divalent guest **2** (with two adamantane units and a single mannose) the maximum surface density of mannose is only half of the surface density that can be achieved with monovalent guest **1**. As a consequence, the average distance *d*_CH_ of the mannose residues is similar to the effective binding-site separation of ConA, and agglutination is quite efficient. However, it can be seen that although agglutination can be completely suppressed by replacement with guest **6**, the concentration dependence is completely different from that observed for guest **1**: rather than showing a threshold value, agglutination persists even at a rather low percentage of guest **2** compared to inert guest **6**. In the case of trivalent guest **3** (with three adamantane units and a single mannose) the maximum surface density of mannose on the cyclodexrin vesicle is only one third of the maximum surface density for guest **1**. As a consequence, the average distance of the mannose residues exceeds the effective binding-site separation of ConA, and very little agglutination is observed upon addition of ConA. Thus, it could be said that more adamantane units in the guest molecule in fact diminish the agglutination by ConA, since they result in a substantially lower surface coverage of mannose.

**Figure 8:**
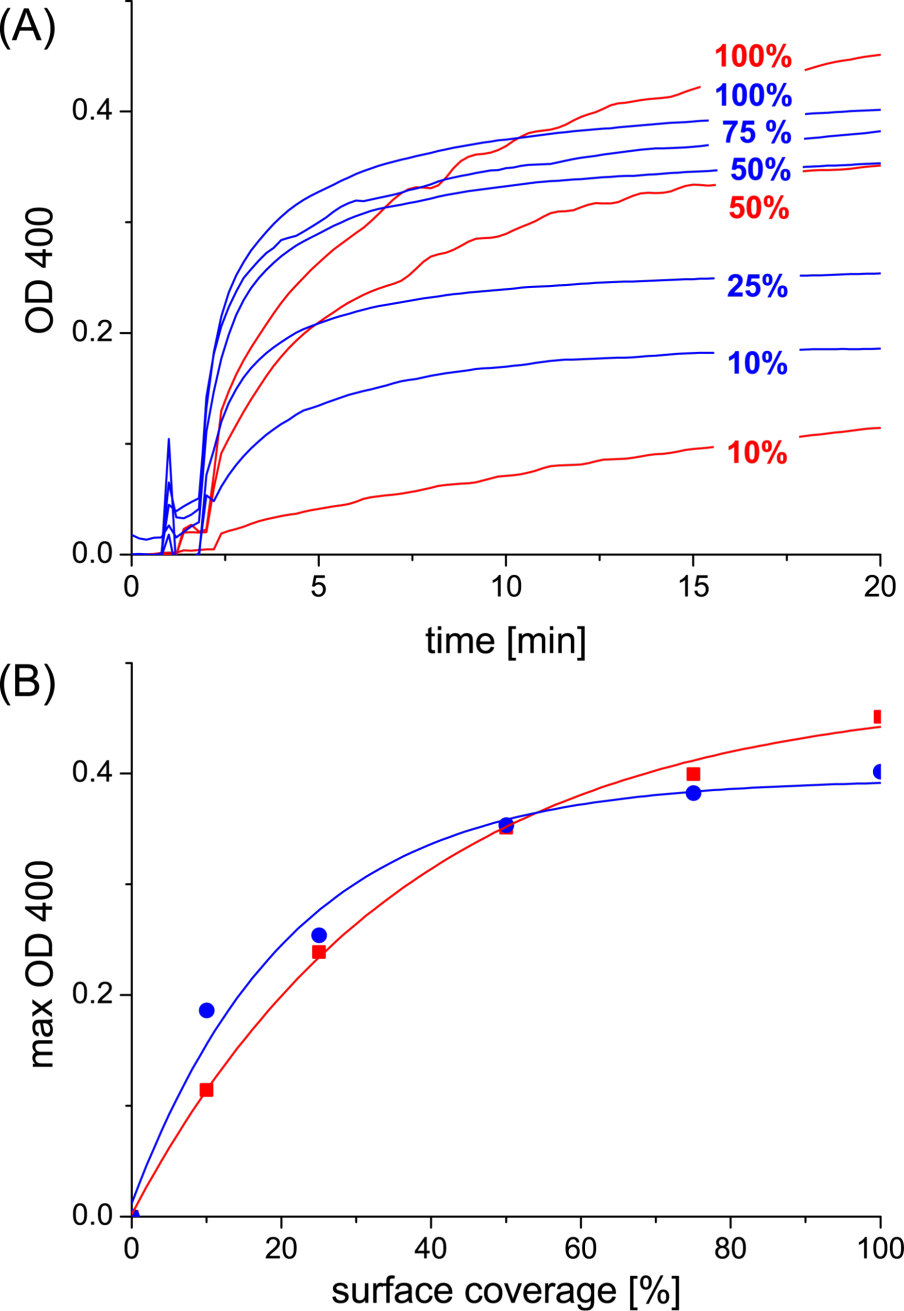
(A) Agglutination of β-cyclodextrin vesicles in the presence of guest **2** or **4** and ConA. Legend: red = guest **2**, blue = guest **4**. The surface coverage of guests **2** and **4** is gradually reduced by substitution with “inert” guest **6**. Concentrations: [5] = 20 μM, [2] = 0–5.0 μM: [4] = 0–5.0 μM, [6] = 0–10 μM; [ConA] = 0.1 mg/mL. (B) Maximal aggregation depending on surface coverage of guest **2** or **4**.

**Figure 9:**
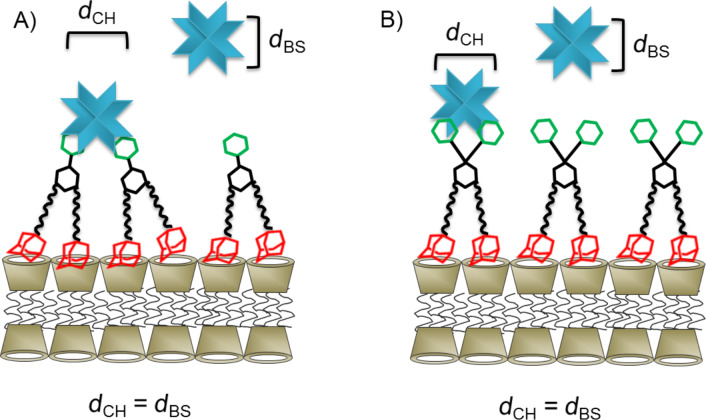
Schematic presentation of the binding of guest molecules with (A) one or (B) two mannose functions onto the surface of cyclodextrin vesicles and their interaction with ConA. *d*_CH_: average spacing of carbohydrates, *d*_BS_: effective binding site separation of ConA.

Finally, we investigated the agglutination behavior of cyclodextrin vesicles decorated with guest **4**. Guest **4** contains two adamantane units as well as two mannose residues. A 100% surface coverage of the cyclodextrin vesicles with guest **4** is therefore expected to give an average distance of 2.2 nm between the mannose functions, similar to the average spacing obtained for guest **1**. However, due to the inherent multivalency of guest **4**, it should have a much higher affinity both for the cyclodextrin vesicle surface as well as for ConA. Indeed it was found that guest **4** ([4] = 10 μM) can induce the agglutination of cyclodextrin vesicles ([5] = 20 μM) in the presence of ConA. Figure 8 shows the results of the optical density measurements for guest **4** compared to guest **2**. It can be seen that the extent and rate of agglutination of the vesicles induced by guest **4** is substantially higher than for guest **2**, in particular at low surface coverage (i.e., below 50 %). At higher surface coverage, the rate of agglutination is much higher for guest **4**, but the extent of agglutination is similar. These observations can be rationalized as illustrated in Figure 9. Since guest **4** has two mannose units, it can bind in a divalent fashion to ConA irrespective of the surface coverage. Nevertheless, the agglutination should still be surface coverage dependent, since multivalent intervesicular binding is more likely to occur when more mannose is presented on the cyclodextrin vesicles. Since guest **2** has only one mannose unit, one would expect that ConA can only bind in a divalent fashion to the cyclodextrin vesicle if the surface coverage is rather high. However, as can be seen from Figure 6 and Figure 8, the extent of agglutination can be very high (as high as for guest **4**) even at rather low surface coverage, albeit with a substantially lower rate of agglutination. These observations can be explained on the basis of a clustering of mannose residues in the mixed glycocalyx of guest **2** and “inert” guest **6**: clusters of guest **2** could offer multivalent “adhesive patches” for ConA even if the average surface coverage of guest **2** is far below 50%. The low rate of agglutination could be a consequence of a slow rearrangement of the glycocalyx, i.e., a “receptor-induced clustering” of mannose in the presence of ConA.

## Conclusion

In this study we described a biomimetic model for the glycocalyx of a cell membrane based on multivalent adamantane–mannose conjugates that bind to cyclodextrin vesicles. In this dynamic supramolecular system the guest molecules bind with their adamantane units to the cyclodextrin cavity, which acts as a receptor, and as a consequence the vesicle surface is covered by mannose. In turn, the mannose units are ligands for the lectin ConA, which induces agglutination due to multivalent cross-linking of the vesicles. Strikingly, the multivalency of the guest molecules was found to have a detrimental effect on the glycocalyx: divalent and trivalent guest molecules can induce cross-linking of the vesicles even in the absence of ConA, and the interaction with ConA is reduced due to the lower surface coverage with mannose. The optimal binder therefore is a divalent guest molecule that carries two mannose residues: this molecule binds inherently divalent to the cyclodextrin vesicle surface as well as to ConA and, hence, is able to mediate fast agglutination at low overall concentration as well as low surface coverage. These findings should further the understanding of the complex and dynamic interactions of oligosaccharides on cell surfaces.

## Experimental

**Materials:** Throughout this work, chemicals were used as received from Acros Organics (Schwerte, Germany) or Sigma-Aldrich Chemie (Taufkirchen, Germany) without further purification. The synthesis and analysis of guest molecule **1–4** is reported in Supporting Information File 1. Amphiphilic β-cyclodextrin **5** was synthesized as described in the literature [30].

**Methods:** Isothermal titration calorimetry (ITC) measurements were performed on a Nano-Isothermal Titration calorimeter III (model CSC 5300; Calorimetry Sciences Corporation, London, Utah, USA). All samples were measured in distilled water at 23 °C by using a stirring rate of 250 rpm. For each experiment 20 injections with 10 µL volume were carried out with a 250 µL syringe, into the measurement cell (*V* = 980.5 µL). Concentrations of host and guest molecules are reported in Table 1. β-Cyclodextrin vesicles are formed by extrusion of cyclodextrin **5** in a HEPES-buffer solution with a Liposofast manual extruder through a polycarbonate membrane with a pore size of 100 nm. Unilamellar vesicles with an average diameter of 100–140 nm are obtained [30,35]. Optical density measurements were performed in 1 mL small-volume disposable PMMA cuvettes at 400 nm by using an Uvikon 923 double-beam photospectrometer. All measurements were recorded at 23 °C in HEPES buffer (20 mM, pH 7.45) containing 1 mM CaCl_2_ and 1 mM MnCl_2_. Reagents were added in the following order: To 1 mL vesicle solution, 10 μL of guest molecule stock solution is added after 1 min, and 10 μL ConA stock solution after 2 min.

## Supporting Information

File 1 Synthesis, NMR and mass spectra of guest molecules **1–4**.
