# Supplementary material for: Mannose-decorated cyclodextrin vesicles: The interplay of multivalency and surface density in lectin–carbohydrate recognition
Source: Beilstein J Org Chem. 2012 Sep 17;8:1543–51. doi: 10.3762/bjoc.8.175 (PMC3510984; doi:10.3762/bjoc.8.175)
Supplement: File 1 — Synthesis, NMR and mass spectra of guest molecules 1–4. [file Beilstein_J_Org_Chem-08-1543-s001.pdf]

Supporting Information

for

Mannose-decorated cyclodextrin vesicles: The interplay of multivalency and surface density in lectin–carbohydrate recognition

Ulrike Kauscher and Bart Jan Ravoo*

Address: Organic Chemistry Institute, Westfälische Wilhelms-Universität Münster, Correnstraße 40, 48149 Münster, Germany

Email: Bart Jan Ravoo* - b.j.ravoo@uni-muenster.de

* Corresponding author

Synthesis, NMR and mass spectra of guest molecules 1–4

Synthesis

General

Throughout this work, chemicals were used as received from Acros Organics (Schwerte, Germany) or Sigma-Aldrich Chemie (Taufkirchen, Germany) without further purification. In the cases where the experimental protocol required an inert gas atmosphere, Schlenk-techniques were carried out under an argon atmosphere. For these techniques the used solvent was dried according to standard methods. Dichloromethane was dried over calcium hydride. Acetone, *N*,*N*-dimethylformamide and methanol were dried by storage over molecular sieves 3Å. Reactions were monitored via thin-layer chromatography (TLC) using 0.2 mm Merck precoated silica gel 60 F254 aluminium sheets. Additional treatment with basic $KMnO_4$ visualized the spots on the plate. If required, column chromatography was carried out on silica gel 60 (0.063–0.2 mm, Merck). NMR spectroscopy was carried out using superconductive Bruker spectrometers (ARX 300, AV 400) and Varian Inova 500, as well as Varian Unity plus 600. Trimethylsilan (δ = 0 ppm) was used as the primary reference, while further references were based on remaining protons in the deuterated solvents. The chemical shift (δ) was measured in parts per million, coupling constants (*J*) were graded in hertz (Hz). Mass Spectrometry was measured on electronspray ionization spectrometers (ESI) *Bruker Daltronics* MicroTof and *Thermo Scientific* Orbi-Trap LTQ-XL with methanol as a solvent. Molecules with higher mass were measured on Matrix assisted laser desorption ionization – time of flight (MALDI-TOF) spectrometry using Lazarus III, *University of Münster*. ITC measurements were recorded on a Nano-Isothermal Titration calorimeter III CSC 5300 by Calorimetry Sciences Corporation. All samples were measured in distilled water at 23 °C using a stirring rate of 250 rpm. For each experiment 20 injections with 10 µL volume were done with a 250 µL syringe into the measurement cell (V = 980.5 µL). DLS was recorded on *Malvern Instruments Ltd.* Nano Zetasizer. The sizes of particles were measured in low volume

disposable PMMA cuvettes while zeta potential was graded in disposable capillary cells. Water was used as an eluent. *Malvern* Dispersion Technology Software was used to analyze data.

Amphiphilic β-cyclodextrin **4** was synthesized as described in literature.[1]

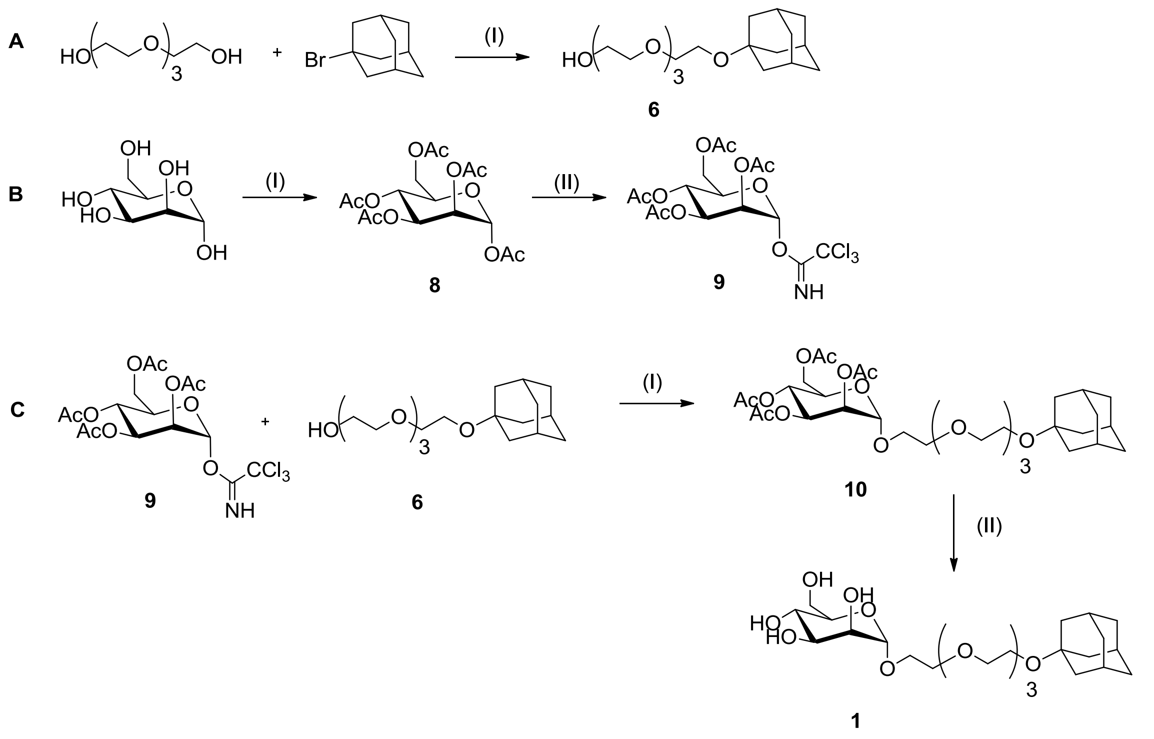

Figure S1: Synthesis of mannose-adamantane-conjugate **1**.A) (I) NEt_3, 180 °C, B) (I) NaOAc, Ac_2O, 80 °C, 2h, (1-3b) = 40% (II) DMF, hydrazine acetate, 2h, 60 °C, (1c) = 46%, C) (I), TMSOTf, CH_2Cl_2, MS 4Å, −25 °C, 25%, (II) NaOMe, MeOH, rt, 97%.

2-(2-{2-[2-(Adamantan-1-yloxy)ethoxy]ethoxy}ethoxy)ethanol (6)[2]

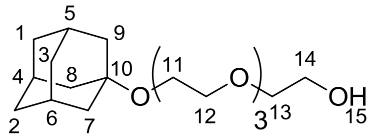

1-Bromoadamantane (16 g, 74.45 mmol) and triethylamine (30 mL, 216 mmol) were dissolved in tetraethylene glycol (270 mL). The solution was stirred overnight at 180 °C. Afterwards it was cooled down to room temperature and dichloromethane (250 mL) was added. The mixture was washed four times with 2M hydrochloride acid (100 mL) and one time with brine (100 mL). The organic layer was dried over $MgSO_4$, evaporated off and a brown oil was obtained (22.33 g, 68.0 mmol, 91%).

Molecular formula: $C_{14}H_{24}O_3$.

^{1}H NMR (300 MHz, $CDCl_3$, 298 K): δ = 1.53-1.63 (m, 6H, 1,2,3-H), 1.71-1.72 (m, 6H, 7,8,9-H), 2.11 (m, 3H, 4,5,6-H), 2.87 (s, 1H, 15-H), 3.53-3.71 (m, 16H, 11-14-H) ppm.

^{13}C NMR (75.5 MHz, $CDCl_3$, 298 K): δ = 30.55 (CH_2, 12-C), 36.51 (CH_2, 13-C), 41.50 (CH_2, 11-C), 59.32 (CH_2, 8-C), 61.79 (CH_2, 1-C), 70.43, 70.64, 70.66, 70.69 (CH_2, 3CH, 3-,4-,5-,6-C), 71.34 (CH_2, 2-C), 72.36 (CH_2, 7-C), 72.62 (CH_2, 9-C) ppm.

HRMS (*m/z*): calculated for $[C_{18}H_{32}O_5Na]^+$: 351.2142, found: 351.2139.

α-D-mannopyranose pentaacetate (7)

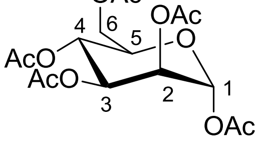

D-Mannose (8 g, 44.4 mmol) and sodium acetate (4.00 g, 48.8 mmol) were dissolved in acetic anhydride (40.0 g, 391 mmol). The reaction mixture was heated under reflux conditions until complete solvation. Afterwards it stayed under reflux conditions for 10 minutes and was then cooled to room temperature. Dichloromethane (250 mL) was added and the organic layer was

washed three times with sodium carbonate solution (100 mL) and dried over $MgSO_4$. The solvent was removed under reduced pressure to yield brown oil (6.90 g, 17.7 mmol, 40%).

Molecular formula: $C_{16}H_{22}O_{11}$.

^{1}H NMR (300 MHz, $CDCl_3$, 298 K): δ = 1.87-2.23 (m, 15H, OAc), 4.03-4.29 (m, 3H, 5,6-H), 5.05-5.43 (m, 3H, 2,3,4-H), 6.02 (s, 1H, 1-H) ppm.

^{13}C NMR (75.5 MHz, $CDCl_3$, 298 K): δ = 20.63, 20.73, 20.75, 20.80, 20.85, 20.86 (5 CH_3, OAc), 168.15, 169.62, 169.83, 170.27, 170.72 (5 C_q, OAc) ppm.

HRMS (*m/z*): calculated for $[C_{16}H_{22}O_{11}Na]^+$: 413.11, found: 413.10.

2,3,4,6-Tetra-O-acetyl-α-D-mannopyranosyl trichloroacetimidate (8)[5]

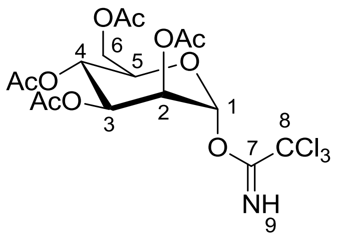

D-Mannose pentaacetate **7** (6.91 g, 17.6 mmol) and hydrazine acetate (1.95 g, 21.18 mmol) were stirred in dry dimethylformamide (40 mL) for 2h at 60 °C under an argon atmosphere. Afterwards the reaction mixture was diluted with ethyl acetate (20 mL) before being washed with distilled water (10 mL) and brine (10 mL). The organic layer was dried over $MgSO_4$ and the solvent was evaporated. The residue was then dissolved in dry dichloromethane (10 mL). Trichloroacetonitrile (17.66 mL, 25.44 g, 176 mmol) was added and the solution was cooled to 0 °C. 1,8-Diazabicyclo[5.4.0]undec-7-ene (263 µL, 268 mg, 1.76 mmol) was added and the mixture was stirred for 1h at 0 °C. It was additionally stirred for 2h at room temperature. The solvent was evaporated off and column chromatography (cyclohexane -EtOAc (2:1 to 1:1), R_f: 0.25) was applied to obtain the product as a yellow white oil (4.02 g, 8.18 mmol, 46%).

Molecular formula: $C_{16}H_{20}Cl_3NO_{10}$.

^{1}H NMR (300 MHz, $CDCl_3$, 298 K):δ = 1.99-2.19 (m, 12H, OAc), 4.12-4.29 (m, 4H, 2,5,6-H), 5.37-5.40 (m, 2H, 3,4-H), 6.26 (d, 1H, 1-H), 8.78 (s, 1H, 9-H) ppm.

^{13}C NMR (75.5 MHz, $CDCl_3$, 298 K): δ = 20.56-21.03 (4 CH_3, OAc), 61.84 (CH_2, 6-C), 65.47, 68.08, 69.13, 71.33 (4 CH, 2,3,4,5-H), 90.71 (CH, C-1), 94.76 (C_q, C-8), 159.75 (C_q, 7-C), 169.66-170.71 (C_q, OAc) ppm.

HRMS (*m/z*): calculated for $[C_{16}H_{22}O_{11}Na]^+$: 413.11, found: 413.10.

2-(2-{2-[2-(adamantan-1-yloxy)ethoxy]ethoxy}ethoxy)ethyl 2,3,4,6-Tetra-O-acetyl-α-D-mannopyranosid (9)

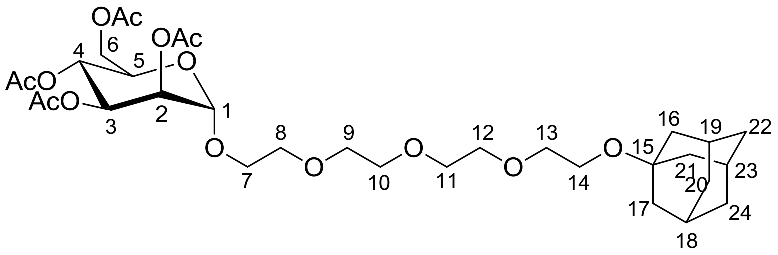

To a solution of compound **8** (1 g, 2.6 mmol) in dry dichloromethane (10 mL) was added compound **6** (0.84 g, 2.6 mmol) under an argon atmosphere. Molecular sieves 4 Å were added and the mixture was cooled to -25 °C. Trimethylsilyl trifluoromethanesulfonate (TMSOTf) (0.24 mL, 0.294 g, 1.32 mmol) was added and the solution was stirred for 2h at -25 °C. The reaction was quenched with $NaHCO_3$ solution and the mixture was extracted with dichloromethane. The organic layer was dried over $MgSO_4$ and the solvents were evaporated off. Column chromatography (Ethylacetate-Pentan (2:1), Rf: 0.29) was applied to yield pure product as a yellow oil (450 mg, 0.628 mmol, 25%).

Molecular formula: $C_{32}H_{50}O_{14}$.

^{1}H NMR (400 MHz, $CDCl_3$, 298 K):δ = 1.54 (q, J = 12.2 Hz, 6H, 20-,22-,24-H), 1.67 (d, J = 2.7 Hz, 6H, 16-,17-,21-H), 2.24 – 1.82 (m, 15H, 18-,19-,23-H, OAc), 3.48-3.82 (m, 16H, 7-14-H), 3.97 - 4.08 (m, 2H, 5-,6-H), 4.19-4.28 (m, 1H, 6-H), 4.79 (d, J = 1.6 Hz, 1H, 1-H), 5.23 (m, 3H, 2-,3-,4-H) ppm.

^{13}C NMR (101 MHz, $CDCl_3$, 298 K): δ = 20.88, 20.84, 20.81, 21.02 (4 CH_3, OAc), 30.61 (3 CH, 18,19,23-C), 36.58 (CH_2, 20,22,24-C), 41.59 (CH_2, 16,17,21-C), 59.35 (CH_2, 14-C), 62.52 (CH_2, 6-C), 66.26 (CH_2, 7-C), 67.50, 68.50, 69.20, 69.69, 70.10, 70.72 (CH2, 8-13-C), 70.73 (CH, 2-C), 70.75 (CH, 3-C), 70.84 (Cq, 15-C), 71.39 (CH, 3-C), 72.32 (CH, 5-C), 97.83 (CH, 1-C), 169.86, 169.97, 170.13, 170.79 (C_q, OAc) ppm.

HRMS (*m/z*): calculated for $[C_{32}H_{50}O_{14}Na]^+$: 681.3093, found: 681.3089.

2-(2-{2-[2-(adamantan-1-yloxy)ethoxy]ethoxy}ethoxy)ethyl α-D-mannopyranosid (1)

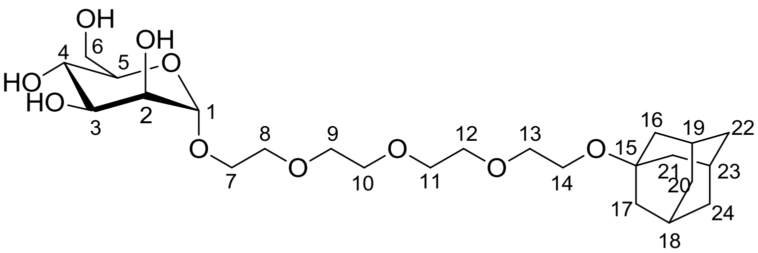

Compound **5** (301 mg, 0.420 mmol) was dissolved in dry methanol (5 mL) under an atmosphere of argon and a solution of NaOMe (catalytic amounts) was added. The reaction was monitored by TLC. By addition of Dowex HCR-W2 in its hydrogen form the solution was neutralized. The mixture was stirred for 15 min and was then filtered. Methanol was evaporated off to yield pure product as a colorless oil (218 mg, 0.445 mmol, 97%).

Molecular formula: $C_{24}H_{42}O_{10}$.

^{1}H NMR (600 MHz, CD_3OD , 298 K): δ = 2.14 (s, 6H, 20-,22-,24-H), 1.78 (s, 6H, 16-,17-,21-H), 1.67 (dd, *J* = 30.2, 12.3 Hz, 3H, 18-,19-,23-H), 3.57-3.72 (m, 19H, 3-,4-,5-,7-14-H), 3.81-3.83 (m, 3H, 2-,6-H), 4.80 (d, *J* = 1.2 Hz, 1H, 1-H) ppm.

^{13}C NMR (151 MHz, CD_3OD, 298 K): δ = 31.96 (CH, 18-,19-,23-C), 37.48 (CH_2, 20-,22-,24-C), 42.52 (CH_2, 16-,17-,21-C), 60.40 (CH_2, 14-C), 62.92 (CH_2, 6-C), 67.75 (CH_2, 7-C), 68.57, 71.37, 71.55, 71.59 (CH_2, 8-13-C), 72.08 (CH, 2-C), 72.18 (CH, 3-C), 72.53 (Cq, 15-C), 73.65 (CH, 3-C), 74.58 (CH, 5-C), 101.72 (CH, 1-C) ppm.

IR (neat) [cm^{-1}]: ν = 679 (w), 813 (w), 880 (w), 978 (m), 1032 (s), 1086 (s), 1133 (s), 1247 (m), 1035 (m), 1355 (m), 1454 (m), 1636 (w), 1530 (w), 1636 (w), 1726 (w), 2394 (w), 2852 (s), 2907 (s), 3425 (br).

HRMS (*m/z*): calculated for $[C_{24}H_{42}O_{10}Na]^+$: 513. 2670, found: 513.2669.

Specific rotation: $[\alpha]_D^{20}$ = 53 (MeOH).

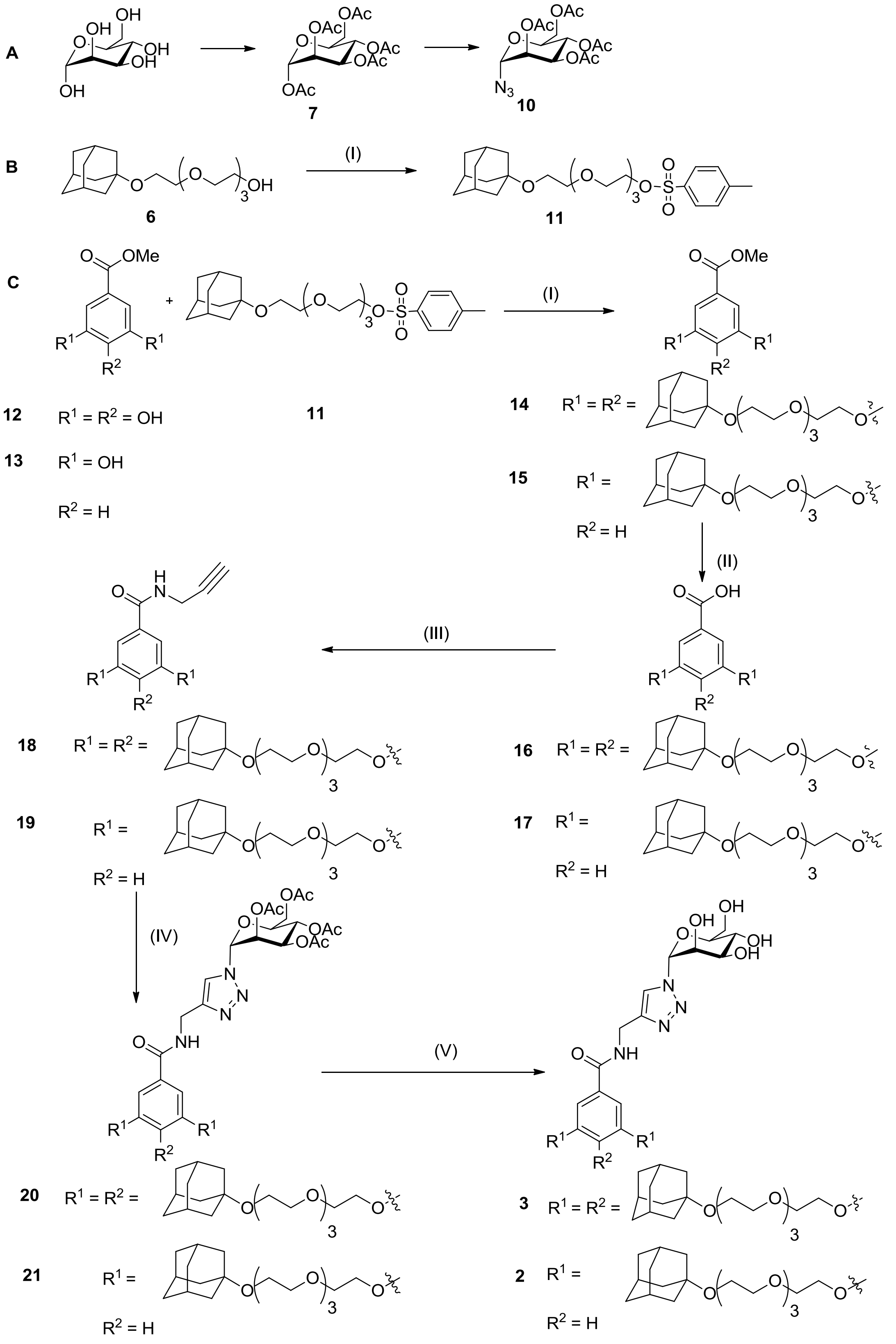

Figure S2: Synthesis of mannose-adamantane-conjugates **2** and **3**. A) (I) NaOAc, Ac_2O, 80 °C, 2h, (1-3b) = 40% (II) $SnCl_4$, trimethylsilylazide, $CHCl_3$, rt, 12 h, (2/3c) = 95%, B) DMAP, NEt_3, TosCl, 0 °C, 12h, (2/3d) = 77%, C) (I) K_2CO_3, acetone, 48 h, 70 °C, (13) = 15%, (12) = 52%,(II) LiOH, H_2O,

THF, MeOH, 12 h, rt, (15) = 87%, (14) = 88%, (III) $NH_2CH_2CCH_2$, EDCI, NMM, HOBt, DMF, 24h, rt, (17) = 95%, (16) = 60%, (IV) mannosemonoazide, CuI, DMF, 60 °C, 24 h, (19) = 15%, (18) = 76%, (V) MeOH, NaOMe, rt, (3) = 75%, (2) = 88%.

2,3,4,6-Tetra-O-acetyl-α-D-mannopyranosyl monoazide (10)

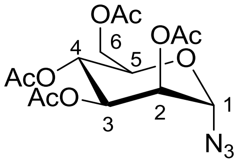

D-Mannose-peracetate **7** (1.00 g, 2.56 mmol), $SnCl_4$ (801 mg, 3.08 mmol, 1.2eq) and trimethylsilyl azide (355 mg, 3.08 mmol) were dissolved in chloroform (10 mL). The solution was stirred overnight at room temperature and afterwards washed with distilled water (10 mL), brine (10 mL) and again water (10 mL). The organic layer was dried over $MgSO_4$ and the solvent was evaporated off to give the product as a brown solid (910 mg, 2.44 mmol, 95%).

Molecular formula: $C_{14}H_{19}N_3O_9$.

^{1}H NMR (300 MHz, $CDCl_3$, 298 K): δ = 1.95-2.18 (m, 12H, OAc), 4.03-4.22 (m, 2H, 4-,5-H), 4.30 (dd, J = 12.5, 5.6 Hz,1H, 2-H), 5.00-5.33 (m, 3H,3-,6-H), 5.38 (d, J = 1.9 Hz, 1H, 1-H) ppm.

^{13}C NMR (101 MHz, $CDCl_3$, 298 K): δ = 20.72, 20.78, 20.82, 20.93 (4 CH_3,OAc), 62.25 (CH_2, 6-C), 65.71 (CH, 3-C), 68.35 (CH, 2-C), 69.28 (CH, 4-C), 70.73 (CH, 5-C), 87.57 (CH, 1-C), 169.76, 169.88, 169.99, 170.73 (5 C_q, OAc) ppm.

HRMS (*m/z*): calculated for $[C_{14}H_{19}N_3O_9Na]^+$: 396.1014, found: 396.1020.

2-(2-{2-[2-(Adamantan-1-yloxy)ethoxy]ethoxy}ethoxy)ethyl monotosylate (11)

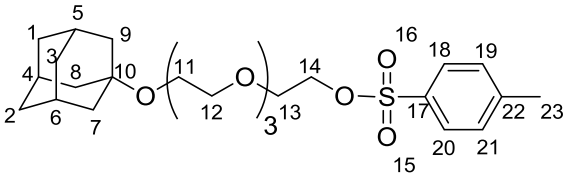

1-Tetraethyleneglycoladamantane **6** (5 g, 15.1 mmol) and 4-(dimethylamino)-pyridin (100 mg, cat.) were dissolved in dichloromethane (250 mL). At 0 °C triethylamine (3.3 mL, 23.0 mmol) and then tosylchloride (3.17 g, 16.6 mmol) was added dropwise. The solution was stirred overnight. Afterwards it was washed two times with water (100 mL), then one time with 2M hydrochloride acid (100 mL) and dried over $MgSO_4$. The solvents were evaporated to yield a yellow-brown oil (6.02 g, 11.92 mmol, 79%).

Molecular formula: $C_{25}H_{38}O_7S$.

^{1}H NMR (300 MHz, $CDCl_3$, 298 K): δ = 1.49-1.59 (m, 6H, 1,2,3-H), 1.66-1.67 (m, 6H, 7,8,9-H), 2.05 (bs, 3H, 4,5,6-H), 2.38 (bs, 3H, 23-H), 3.478-3.63 (m, 14H , 11-13-H), 4.07-4.11 (tr, 2H, 14-H), 7.27 (d, J = 8Hz, 2H, 19,21-H), 7.72 (d, J = 8.9 Hz, 2H, 18,20-H) ppm.

^{13}C NMR (75.5 MHz, $CDCl_3$, 298 K): δ = 21.55 (CH_3, 23-C), 30.53 (CH, 4,5,6-H), 36.49 (CH_2, 1-,2-,3-H), 41.52 (3 CH_2, 7-,8-,9-H), 68.28, 68.89, 70.14, 70.19, 70.25, 70.36, 70.89, 71.83 (8 CH_2, 11,12,13,14-H), 77.16 (C_q, 10-C), 127.08, 128.02, 130.30, 133.05 (4 CH, 18-21-H), 141.70 (C_q, 17-C), 146.84 (C_q, 22-C) ppm.

HRMS (*m/z*): calculated for $[C_{25}H_{38}O_7SNa]^+$: 505.22, found: 505.2224.

3,4,5-Tris[2-(2-{2-[2-(adamantan-1-yloxy)ethoxy]ethoxy}ethoxy)ethoxy]benzoic acid methyl ester (12)[3]

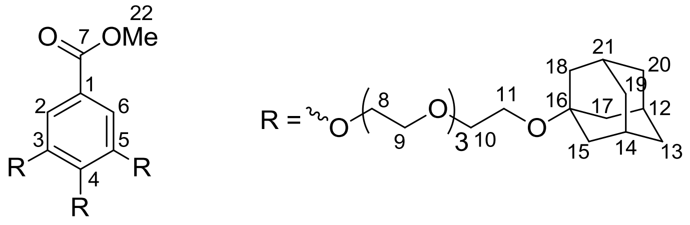

Trihydroxybenzoate methylester (0.43 g, 2.36 mmol) was dissolved in dry acetone. Potassium carbonate (3.26 g, 23.6 mmol) and **11** (3 g, 8.27 mmol) was added. The reaction was stirred for 48h under reflux conditions and an argon atmosphere. The pink reaction mixture was filtered to remove potassium carbonate. The solvent was evaporated and the crude product was redissolved in dichloromethane and was washed with water (50 mL), 1M HCl (40 mL) and again with water (50 mL). The organic layer was dried over $MgSO_4$ and the solvents were removed under reduced pressure. Column chromatography (SiO_2: dichloromethane-methanol, (95:5), Rf = 0.29) was applied to give pure product as a brown oil (300 mg, 0.353 mmol, 15%).

Molecular formula: $C_{62}H_{98}O_{17}$.

^{1}H NMR (300 MHz, $CDCl_3$, 298 K): δ = 1.59 (t, J = 8.9 Hz, 18H, 13-,19-,20-H), 1.70 (d, *J* = 8.9 Hz, 18H, 15-,17-,18-H), 2.10 (bs, 9H, 12-,14-,21-H), 3.36-3.84 (m, 42H, 9-11-H), 3.85 (s, 3H, 22-H), 4.00 – 4.34 (m, 6H, 8-H), 7.26 (s, 2H, 2-,6-H) ppm.

^{13}C NMR (75 MHz, $CDCl_3$, 298 K): δ = 30.48 (9 CH, 12-,14-,21-C), 36.44 (9 CH_2, 13-,19-,20-C), 41.45 (9 CH_2, 15-,17-,18-C), 52.15 (CH_3, 22-C), 59.23 (3 CH_2, 11-C), 68.82, 69.59, 70.25, 70.55, 70.63, 70.81, 71.26 (24 CH_2, 8-,9-,10-C) , 72.22 (3 C_q, 16 C), 109.01 (2 CH, 2-,6-C), 124.93 (C_q, 1-C), 146.53 (C_q, 4-C), 152.27 (C_q, 3-,5-C), 166.61 (C_q, 7-C) ppm.

HRMS (*m/z*): calculated for $[C_{62}H_{98}O_{17}Na]^+$: 1137.6696, found: 1137.6693.

3,5-Bis[2-(2-{2-[2-(adamantan-1-yloxy)ethoxy]ethoxy}ethoxy)ethoxy]benzoic acid methyl ester(13)

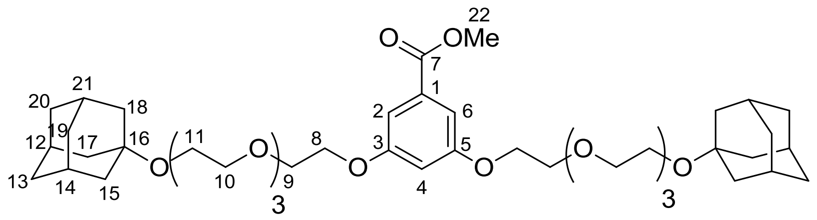

Synthesis was carried out as described for compound **12**. Dihydroxybenzoate methylester (0.501 g, 2.98 mmol) was dissolved in dry acetone together with compound **11** (2.6 g, 7.167 mmol). The product was obtained in the form of a yellow oil (1.22 g, 1.55 mmol, 52%).

Molecular formula: $C_{44}H_{68}O_{12}$.

^{1}H NMR (300 MHz, $CDCl_3$, 298 K): δ = 1.59 (p, *J* = 12.8 Hz, 12H, 13-,19-,20-H), 1.72 (d, *J* = 2.8 Hz, 12H, 15-,17,18-H), 2.12 (s, 6H, 12-,14-,21-H), 3.50 – 3.75 (m, 24H, 10-11-H), 3.78-3.86 (m, 4H, 9-H), 3.88 (s, 3H, 22-H) 4.02-4.19 (m, 4H, 8-H), 6.68 (t, *J* = 2.3 Hz, 1H, 4-H), 7.18 (d, *J* = 2.3 Hz, 2H, 2-,6-H) ppm.

^{13}C NMR (75.5 MHz, $CDCl_3$, 298 K): δ = 30.61 (3 CH, 12-,14-,21-C), 36.57 (3 CH_2, 13-,19-,20-C), 41.58 (3 CH_2, 15-,17-,18-C), 52.32 (CH_3, 22-C), 59.36 (2 CH_2, 11-C), 67.86, 69.70, 70.72, 70.76, 70.77, 70.97, 71.39 (14 CH_2, 8-10-C), 72.33 (2 C_q, 16-C), 106.99 (2 CH, 2-,6-C), 108.13 (CH, 4-C), 131.98 (C_q, 1-C), 159.87 (2 C_q, 3-,5-C), 166.87 (C_q, 7-C) ppm.

HRMS (*m/z*): calculated for $[C_{44}H_{68}O_{12}Na]^+$: 811.4603, found: 811.4616.

3,4,5-Tris[2-(2-{2-[2-(adamantan-1-yloxy)ethoxy]ethoxy}ethoxy)ethoxy]benzoic acid (14)

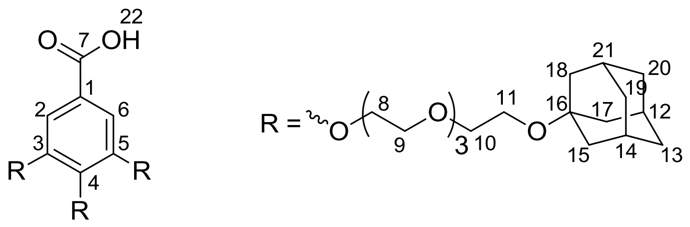

Compound **12** (168 mg, 0.15 mmol) was added to a solution of lithium hydroxide (16 mg, 0.67 mmol) in water/methanol (3 mL). The mixture was stirred for two days. After this period the solvents were evaporated off and residue was redissolved in dichloromethane (30 mL) and washed with water (20 mL), with citric acid (20 mL) and with brine (20 mL). The organic layer was dried over $MgSO_4$ and the solvents were evaporated off to yield pure product as a yellow oil (147 mg, 0.13 mmol, 87%).

Molecular formula: $C_{61}H_{96}O_{17}$.

^{1}H NMR (300 MHz, $CDCl_3$, 298 K): δ = 1.59 (q, J = 12.4 Hz, 18H, 13-,19-,20-H), 1.72 (t, J = 11.4 Hz, 18H, 15-,17-,18-H), 2.17 – 2.07 (m, 9H, 12-,14-,21-H), 3.93 – 3.40 (m, 45H, 9-11-H), 4.27 – 4.14 (m, 6H, 8-H), 7.36 (s, 2H, 2-,6-H) ppm.

^{13}C NMR (75.5 MHz, $CDCl_3$, 298 K): δ = 30.62 (3 CH, 12-,14-,21-C), 36.55 (3 CH_2, 13-,29-,20-C), 41.53 (3 CH_2, 15-,17-,18-C), 59.40 (3 CH_2, 11-C), 69.01, 69.88, 70.70, 70.77, 70.80, 70.98, 71.34 (24 CH_2, 8-,9-,10-C), 72.67(3 C_q, 16 C), 109.85 (2 CH, 2-,6-H), 124.70 (C_q, 1-C), 143.09 (C_q, 4-C), 152.41 (2 C_q, 3-5-C), 169.17 (C_q, 7-C) ppm.

HRMS (m/z): calculated for $[C_{61}H_{96}O_{17}Na]^+$: 1123.6540, found: 1123.6525.

3,5-Bis[2-(2-{2-[2-(adamantan-1-yloxy)ethoxy]ethoxy}ethoxy)ethoxy]benzoic acid (15)

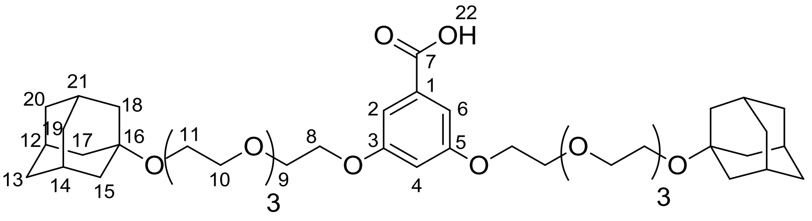

Synthesis was carried out as described for compound **14**. Compound **13** (1.637 g, 2.08 mmol) was used as starting material. Product was obtained as a yellow oil (1.38 g, 1.84 mmol, 88%).

Molecular formula: $C_{43}H_{64}O_{12}$.

^{1}H NMR (300 MHz, $CDCl_3$, 298 K): δ =1.47–1.66 (m, 12H, 13-,19-,20-H), 1.74 (d, *J* = 2.8 Hz, 12H, 15-,17-,18-H), 2.12 (s, 6H, 12-,14-,21-H), 3.78 – 3.63 (m, 24H, 10-11-H),), 3.84 (dd, *J* = 9.3, 5.3 Hz, 4H, 9-H), 4.21 – 4.02 (m, 4H, 8-H), 6.70 (t, *J* = 2.3 Hz, 1H, 4-H), 7.23 (d, *J* = 2.3 Hz, 2H, 2-,6-H) ppm.

^{13}C NMR (75.5 MHz, $CDCl_3$, 298 K): δ =30.62 (3 CH, 12-,14-,21-C), 36.56 (3 CH_2, 13-,19-,20-C), 41.55 (3 CH_2, 15-,17-,18-C), 59.39 (2 CH_2, 11-C), 67.88, 69.77, 70.70, 70.76, 70.96, 71.35 (14 CH_2, 8-10-C), 72.59 (2 C_q, 16-C), 107.68 (2 CH, 2-,6-C), 108.62 (CH, 4-C), 130.04 (C_q, 1-C), 159.90 (2 C_q, 3-,5-C), 170.07 (C_q, 7-C) ppm.

HRMS (*m/z*): calculated for $[C_{43}H_{65}O_{12}]^+$: 773.4482, found: 773.4501.

<u>*N*-(prop-2-yn-1-yl)-3,4,5-tris[2-(2-{2-[2-(adamantan-1-yloxy)ethoxy]ethoxy}ethoxy) ethoxy]benzyamide (16)</u>

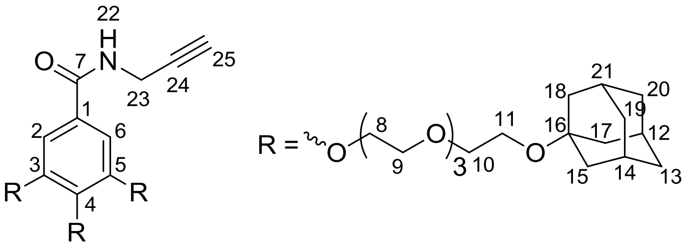

A name could not be generated for this structure.

Compound **14** (1.00 g, 0.91 mmol), 1-ethyl-3-(3-dimethylaminopropyl)carbodiimide (261 mg, 1.36 mmol) and hydroxybenzotriazole (184 mg, 1.36 mmol) were dissolved in dimethylformamide and were stirred for 30 minutes. Afterwards propargylamine (74.9 mg, 1.36 mmol) and N-methylmorpholine (138 mg, 1.36 mmol) were added and the mixture was stirred overnight at room temperature. After this period the solvents were evaporated and the residue was redissolved in chloroform. The solution was washed with brine, distilled-water, $NaHCO_3$ solution and again with distilled water. The organic layer was dried over $MgSO_4$ and the solvents were evaporated off to give crude product, which was then purified by column chromatography (SiO_2: ethylacetate, R_f: 0.41) to yield a yellow oil (1.00 g, 0.86 mmol, 95%).

<u>Molecular formula:</u> $C_{64}H_{99}NO_{16}$.

<u>^{1}H NMR (300 MHz, $CDCl_3$, 298 K):</u>δ = 1.54 (s, 18H, 13-,19-,20-H), 1.67 (d, *J* = 5.0 Hz, 18H, 15-,17-,18-H), 2.06 (s, 9H, 12-,14-,21-H), 2.18 (d, *J* = 2.5 Hz, 1H, 25-H), 3.47-3.64 (m, 42H, 9-11-H),), 4.15 (d, *J* = 3.3 Hz, 8H, 8-,23-H), 7.13 (s, 1H, 22-H) ,7.95 (s, 2H, 2-,6-H) ppm.

<u>^{13}C NMR (75 MHz, $CDCl_3$, 298 K):</u> δ = 29.64 (CH2, 23-C), 30.55 (3 CH, 12-,14-,21-C), 36.50 (3 CH2, 13-,29-,20-C), 41.48 (3 CH2, 15-,17-,18-C), 59.29 (3 CH2, 11-C), 69.83, 70.23, 70.61, 70.72, 71.33, 72.44, 72.66 (21 CH2, 8-,9-,10-C), 72.88 (3 Cq, 16 C), 86.81 (CH, 25-C), 86.97 (Cq, 24-C), 116.58 (2 CH, 2-,6-C), 146.90 (Cq, 1-C), 152.44 (CH, 4-C), 162.65 (2 Cq, 3-,5-C), 164.83 (Cq, 7-C) ppm.

HRMS (*m/z*): calculated for $[C_{64}H_{99}NO_{16}Na]^{+}$: 1160.6862, found: 1160.6853.

_N_-(prop-2-yn-1-yl)-3,5-Bis[2-(2-{2-[2-(adamantan-1-yloxy)ethoxy]ethoxy}ethoxy) ethoxy]benzamide (17)

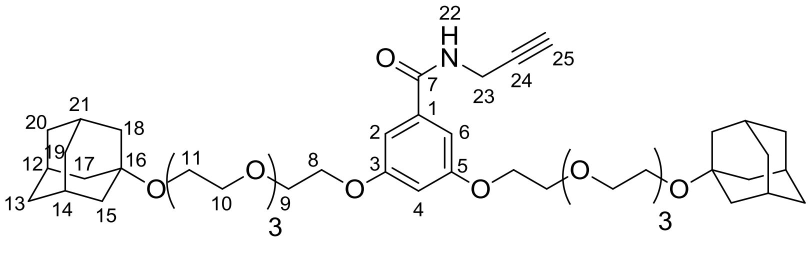

Synthesis was carried out as described for compound **16**. Compound **15** (628 mg, 0.81 mmol) was used as starting material. The crude product was in the end purified by column chromatography (SiO_2: ethylacetate, R_f: 0.57). A yellow oil was obtained (407 mg, 0.49 mmol, 60%).

Molecular formula: $C_{46}H_{69}NO_{11}$

^{1}H NMR (300 MHz, $CDCl_3$, 298 K): δ = 1.49-1.66 (m, 12H, 13-,19-,20-H), 1.70 (d, *J* = 2.7 Hz, 12H, 15-,17-,18-H), 2.10 (s, 6H, 12-,14-,21-H), 2.25 (s, 1H, 25-H), 3.41-3.75 (m, 24H, 10-11-H), 3.77-3.90 (m, 4H, 9-H), 4.05-4.15 (m, 4H, 8-H), 4.19 (d, *J* = 2.7 Hz, 2H, 23-H), 6.88 (t, *J* = 5.2 Hz, 1H, 4-H), 6.58 (s, 1H, 22-H), 6.96 (d, *J* = 2.2 Hz, 2H, 2-,6-H) ppm.

^{13}C NMR (75.5 MHz, $CDCl_3$, 298 K): δ = 29.77 (CH_2, 23-C), 30.54 (3 CH, 12-,14-,21-C), 36.49 (3 CH_2, 13-,19-,20-C), 41.49 (3 CH_2, 15-,17-,18-C), 59.29 (2 CH_2, 11-C), 67.79, 69.69, 70.62, 70.66, 70.85, 71.31, 71.67 (14 CH_2, 8-10-C), 72.42 (2 C_q, 16-C), 78.17 (CH, 25-C), 79.80 (C_q, 24-C), 105.30 (2 CH, 2-,6-C), 106.09 (CH, 4-C), 135.85(C_q, 1-C), 159.96 (2 C_q, 3-,5-C), 166.93 (C_q, 7-C) ppm.

HRMS (*m/z*): calculated for $[C_{46}H_{69}NO_{11}Na]^{+}$: 834.4763, found: 834.4768.

N-{[1-(2,3,4,6-tetra-O-acetyl-α-D-mannopyranosyl)-1H-1,2,3-triazol-4-yl]methyl}-3,4,5-tris[2-(2-{2-[2-(adamantan-1-yloxy)ethoxy]ethoxy}ethoxy)ethoxy]benzamide (18)

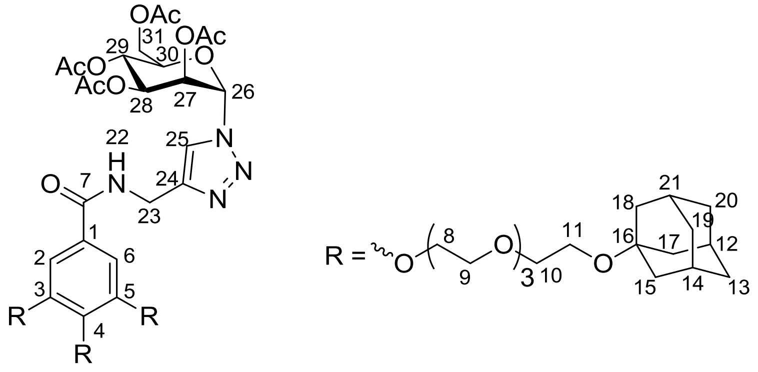

Compound **17** (379 mg, 0.33 mmol) and compound **10** (150 mg, 0.40 mmol) were dissolved in dimethylformamide. Catalytic amounts of copper(I)iodide were added and the suspension was stirred at 60 °C overnight. Afterwards the solvent was evaporated and the mixture was redissolved in chloroform. The solution was washed with brine and water. The organic layer was then dried over $MgSO_4$ and the solvents were again evaporated off. The given crude product was purified via column chromatography (SiO_2: dichloromethane-methanol, (9:1), R_f: 0.41). A brown solid was obtained (40 mg, 0.05 mmol, 15%).

Molecular formula: $C_{78}H_{118}N_4O_{25}$

^{1}H NMR (400 MHz, $CDCl_3$, 298 K):δ = 1.41-1.68 (m, 18H, 13-,19-,20-H), 1.71 (dd, *J* = 9.2, 2.7 Hz, 18H, 15-,17-,18-H), 1.86-2.34 (m, 21H, 12-,14-,21-H, OAc), 2.95-4.43 (m, 47H, 8-11-H, 31-H), 4.56 -4.69 (m, 1H, 30-H), 4.69-4.85 (m, 1H, 29-H), 5.20-5.43 (m, 1H, 28-H), 5.87 (m, 1H, 27-H), 5.95 (t, *J* = 4.2 Hz, 1H, 26-H), 7.16 (d, *J* = 13.9 Hz, 2H, 2-,6-H), 7.62 (dd, *J* = 15.7, 10.0 Hz, 1H, 23-H), 7.80-7.91 (m, 1H, 25-H) ppm.

^{13}C NMR (75.5 MHz, $CDCl_3$, 298 K): δ = 20.80 (4 CH_3, OAc), 30.58 (9 CH, 12-,14-,21-C), 36.53(9 CH_2, 13-,19-,20-C), 41.53 (9 CH2, 15-,17-,18-C), 59.32, 59.34 (3 CH_2, 11-C), 61.68, 61.81 (2 CH, 29-,30-C), 68.43 (C_q, 16-C), 68.93 (CH, 28-C), 69.15, 69.81, 70.33, 70.49, 70.50, 70.54, 70.60, 70.64, 70.71, 70.73, 71.34, 71.37, 72.42, 72.45 (21 CH_2, 8-10-,31-C), 77.36 (CH_2, 23-C), 83.77 (CH, 26-C), 107.57 (2 CH, 2-,6-C), 107.65 (2 CH, 3-,5-C),

123.46 (CH, 25-C), 129.12 (C_q, 1-C), 145.95 (C_q, 24-C), 152.50 (C_q, 4-C), 167.08 (CH, 27-C), 169.47 (C_q, 7-C), 169.77, 170.64, 172.84, 174.08 (C_q, OAc) ppm.

HRMS (*m/z*): calculated for $[C_{78}H_{118}N_4O_{25}]^+$: 1533.7977, found: 1533.7959.

N-{[1-(2,3,4,6-tetra-O-acetyl-α-D-mannopyranosyl)-1*H*-1,2,3-triazol-4-yl]methyl}-3,5-bis[2-(2-{2-[2-(adamantan-1-yloxy)ethoxy]ethoxy}ethoxy)ethoxy]benzamide (19)

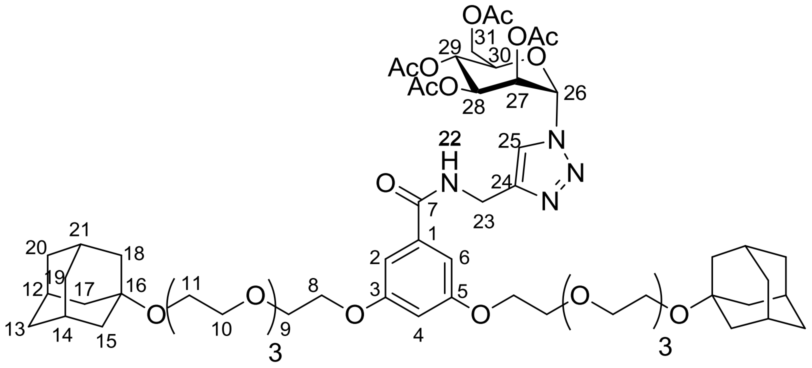

Synthesis was carried out as described for compound **18**. Compound **17** (267 mg, 0.33 mmol) was used as starting material. The crude product was in the end purified by column chromatography (SiO_2: dichloromethane-methanol, (95:5), R_f: 0.82). A yellow oil was obtained (300 mg, 0.25 mmol, 76%).

Molecular formula: $C_{60}H_{88}N_4O_{20}$.

^{1}H NMR (500 MHz, $CDCl_3$, 298 K): δ = 1.58 (m, 12H, 13-,19-,20-H), 1.71 (m, 12H, 15-,17-,18-H), 1.93-2.23 (m, 21H, 12-,14-,21-H, OAc), 3.37-3.95 (m, 29H, 9-11-,29-H), 4.01 (m, 2H, 31-H), 4.18 – 4.05 (m, 4H, 8-H), 4.31 (ddd, *J* = 21.3, 13.5, 7.1 Hz, 1H, 30-H), 4.71 (ddd, *J* = 32.0, 15.0, 7.7 Hz, 2H, 23-H), 5.34 (dt, *J* = 10.4, 3.9 Hz, 1H, 28-H), 5.85-5.94 (m, 1H, 27-H), 5.96 (d, *J* = 2.2 Hz, 1H, 26-H), 6.59 (s, 1H, 4-H), 6.96 (d, *J* = 2.1 Hz, 2H, 2-,6-H), 7.83 (s, 1H, 25-H), 8.00 (s, 1H, 22-H) ppm.

^{13}C NMR (126 MHz, $CDCl_3$, 298 K): δ = 20.86 (4 CH_3, OAc), 30.62 (6 CH, 12-,14-,21-C), 36.58 (6 CH_2, 13-,19-,20-C), 41.59 (6 CH2, 15-,17-,18-C), 59.38, 61.88 (2 CH_2, 11-C), 62.27 (CH, 28-C), 66.16 (C_q, 16-C), 67.94, 68.37 (2 CH, 29-,30-C), 68.91, 69.71, 69.75, 70.70, 70.72, 70.73, 70.75, 70.79, 70.94, 70.98, 71.39, 72.28, 72.42 (21 CH_2, 8-10-C), 77.36 (CH_2, 23-C), 83.81(CH_2, 31-C), 87.58(CH, 26-C), 105.37 (2 CH, 2-,6-C), 106.08 (2 CH, 3-,5-C), 108.15 (CH, 4-C), 123.19 (CH, 25-C), 135.98 (C_q, 1-C), 145.76 (C_q, 24-C), 160.13 (C_q, 7-C), 167.36 (CH, 27-C), 169.45, 169.74, 169.75, 170.63 (C_q, OAc) ppm.

HRMS (*m/z*): calculated for $[C_{60}H_{88}N_4O_{20}Na]^+$: 1207.5884, found: 1207.5847.

N-{[1-(α-D-mannopyranosyl)-1*H*-1,2,3-triazol-4-yl]methyl}-3,4,5-tris[2-(2-{2-[2-(adamantan-1-yloxy)ethoxy]ethoxy}ethoxy)ethoxy]benzamide (3)

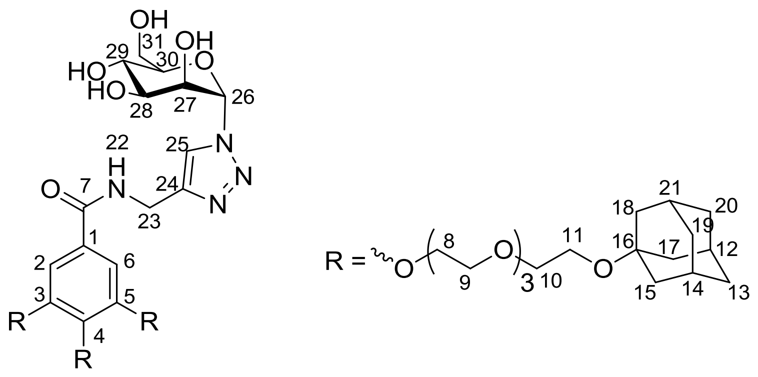

Compound **18** (30 mg, 0.01 mmol) was dissolved in dry methanol and a solution of NaOMe (catalytic amounts) in methanol was added. The mixture was stirred at room temperature until TLC signaled a complete conversion. Afterwards the solution was neutralized by addition of Dowex HCR-W2 ion exchange resign in hydrogen form. The suspension was stirred for 15 minutes and was then filtered. Product was obtained as a colorless waxy solid (20 mg, 0.01 mmol, 75%).

Molecular formula: $C_{70}H_{110}N_4O_{21}$

^{1}H NMR (600 MHz, MeOD, 298 K): δ = 1.57-1.72 (m, 18H, 13-,19-,20-H), 1.72-1.88 (m, 18H, 15-,17-,18-H), 2.09-2.19 (m, 9H, 12-,14-,21-H), 3.34-3.37 (m, 2H, 23-H), 3.48-3.96 (m, 50H, 8-11-,29-,30-H), 4.09-4.11 (dd, *J* = 8.4, 3.6 Hz, 1H, 28-H), 4.23- 4.25 (m, 3H, 22-,31-H), 4.60-4.72 (m, 1H, 27-H), 6.02 (d, *J* = 2.6 Hz, 1H, 26-H), 7.26 (s, 2H, 2-,6-H), 8.12 (s, 1H, 25-H) ppm.

^{13}C NMR (151 MHz, CD_3OD , 298 K): δ = 31.98 (9 CH, 12-,14-,21-C), 37.50 (9 CH_2, 13-,19-,20-C), 42.55 (9 CH2, 15-,17-,18-C), 60.44 (3 CH_2, 11-C), 62.54, 62.23 (2 CH, 29-,30-C), 67.81 (C_q, 16-C), 68.64 (2 CH, 28-C), 70.10, 70.15, 70.81, 71.40, 71.54, 71.57, 71.60, 71.68, 71.73, 71.78, 72.22, 72.59, 73.63, 73.67 (21 CH_2, 8-10-, 31-C), 78.64 (CH_2, 23-C), 88.35 (CH, 26-C), 108.04 (2 CH, 2-,6-C), 116.43 (2 CH, 3-,5-C), 124.48 (CH, 25-C), 130.42 (C_q, 1-C), 142.42 (C_q, 24-C), 153.80(C_q, 4-C), 169.18 (CH, 27-C), 169.20 (C_q, 7-C) ppm.

IR (neat) [cm^{-1}]: ν = 550 (m), 624 (m), 652 (m), 869 (w), 953 (w), 980 (w), 1089 (s), 1107 (s), 1243 (w), 1332 (w), 1453 (w), 1497 (w), 1543 (w), 1582 (w), 1642 (w), 1724 (w), 2852 (m), 2905 (m), 3351 (br).

HRMS (*m/z*): calculated for $[C_{70}H_{110}N_4O_{21}Na]^+$: 1365.7555, found: 1365.7946

Specific rotation: $[\alpha]_D^{20}$ = 56 (MeOH).

_N_-{[1-(α-D-mannopyranosyl)-1_H_-1,2,3-triazol-4-yl]methyl}-3,5-bis[2-(2-{2-[2-(adamantan-1-yloxy)ethoxy]ethoxy}ethoxy)ethoxy]benzamide (2)

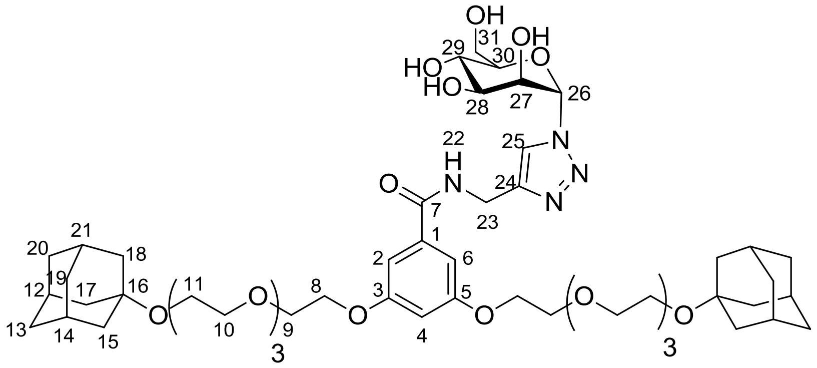

Synthesis was carried out as described for compound **3**. Compound **19** (408 mg, 0.34 mmol) was used as starting material. Product was obtained as a brown wax (300 mg, 0.30 mmol, 88%).

Molecular formula: $C_{52}H_{80}N_4O_{16}$.

^{1}H NMR (500 MHz, MeOD, 298 K): δ = 1.66 (dt, J = 12.5, 6.3 Hz, 12H, 13-,19-,20-H), 1.77 (d, J = 2.4 Hz, 12H, 15-,17-,18-H), 2.09-2.19 (m, 6H, 12-,14-,21-H), 3.55-3.96 (m, 32H, 9-11-, 29-, 30-, 31-H), 4.11 (dd, J = 8.5, 3.5 Hz, 1H, 28-H), 4.15-4.22 (m, 4H, 8-H), 4.69 (m, 2H, 27-H), 6.03 (d, J = 2.7 Hz, 1H, 26-H), 6.73 (t, J = 2.2 Hz, 1H, 4-H),7.07 (d, J = 2.2 Hz, 2H, 2-,6-H), 7.20 (d, J = 2.3 Hz, 1H, 25-H), 8.13 (s, 1H, 22-H) ppm.

^{13}C NMR (126 MHz, MeOD, 298 K): δ = 31.96 (6 CH, 12-,14-,21-C), 37.48 (6 CH_2, 13-,19-,20-C), 42.52 (6 CH_2, 15-,17-,18-C), 60.42 (2 CH_2, 11-C), 62.52 (CH, 28-C), 68.07 (C_q, 16-C), 68.98, 69.03 (2 CH, 29-,30-C), 70.09, 70.74, 71.54, 71.59, 71.61, 71.77, 71.87, 72.20, 72.57, 73.64 (20 CH_2, 8-10-C), 76.97 (CH_2, 31-C), 78.58 (CH_2, 23-C), 88.32 (CH, 26-C), 106.17 (2 CH, 2-,6-C), 107.17 (2 CH, 3-,5-C), 109.05 (CH, 4-C), 124.37 (CH, 25-C), 137.24 (C_q, 1-C), 146.67 (C_q, 24-C), 161.49 (C_q, 7-C), 169.54 (CH, 27-C) ppm.

IR (neat) [cm^{-1}]: ν = 515 (m), 533 (m), 549 (m), 866 (m), 953 (m), 979 (m), 1088 (s), 1106 (s), 1172 (m), 1248 (m), 1304 (m), 1354 (m), 1442 (m), 1537 (m), 1592 (m), 1645 (w), 2113 (w), 2341 (w), 2361 (w), 2852 (s), 2905 (s), 3342 (br), 3410 (br).

HRMS (*m/z*): calculated for $[C_{52}H_{80}N_4O_{16}Na]^+$: 1039.5462, found: 1039.5474.

Specific rotation: $[\alpha]_D^{20}$ = 28 (MeOH).

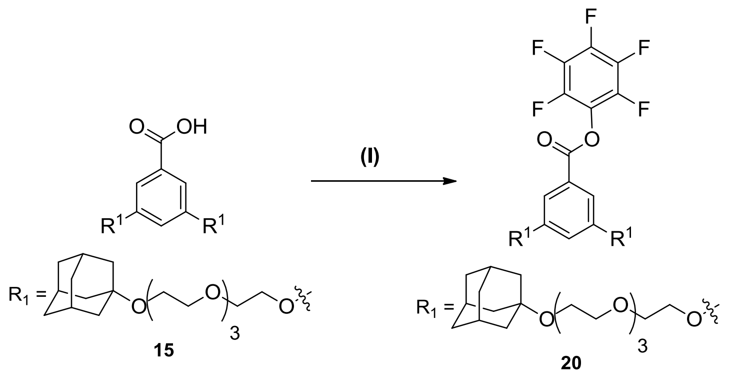

Figure S3: Synthesis route of mannose-adamantane-conjugates **4**. (I) dry DCM, pentafluorophenol, *N*,*N'*-dicyclohexylcarbodiimide, 0 °C, 12h, (20) = 79%.

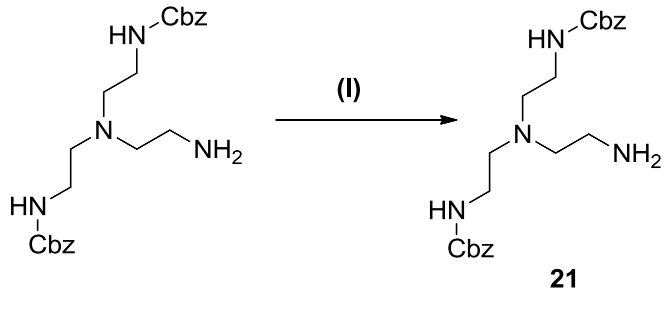

Figure S4: Synthesis route of mannose-adamantane-conjugates **4**. (I) abs. ethanol, benzyl phenyl carbonate, 0 °C, 12h, (21) = 66%.

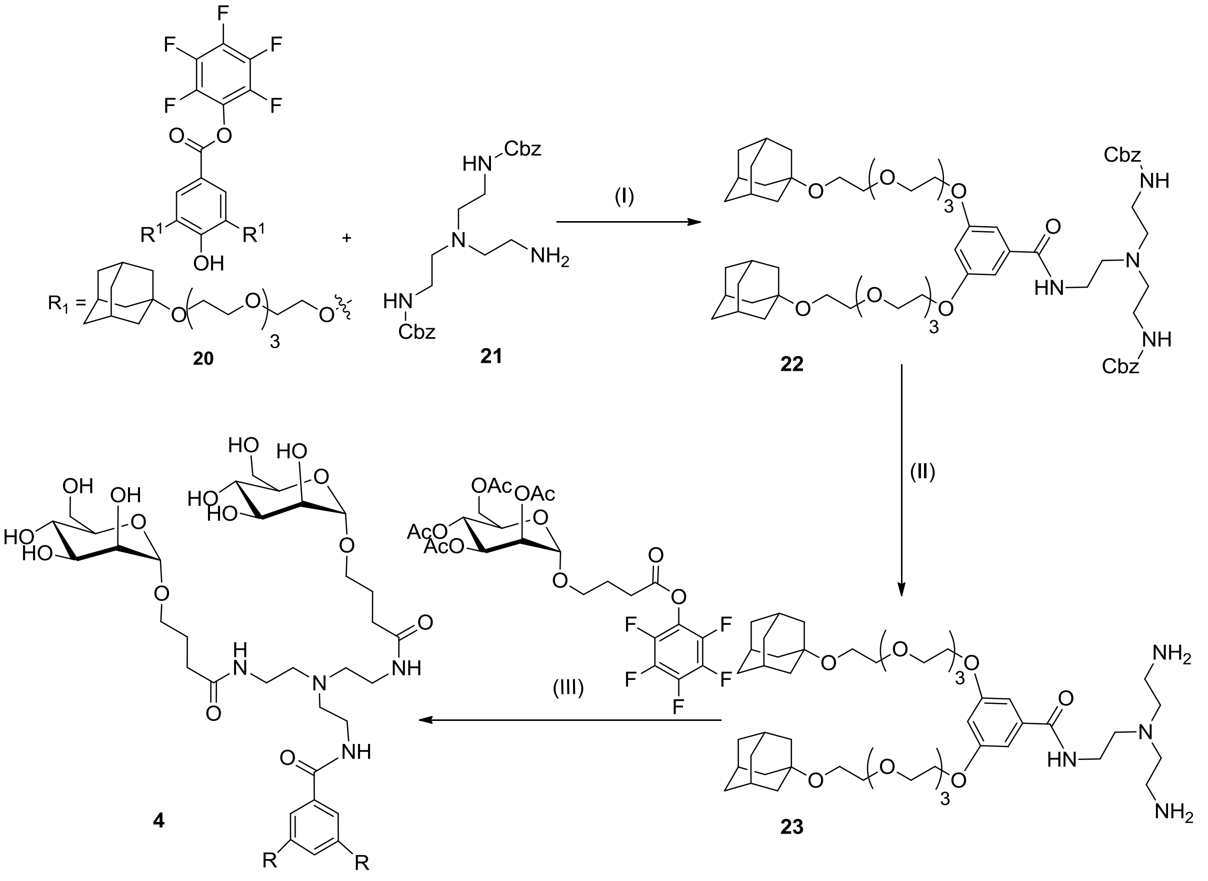

Figure S5: Synthesis of mannose-adamantane-conjugates **4**. (I) EDCI, NMM, DMF, HOBt, 24h, rt, (22)= 72% II) Pd/C, $H_{2,}$ MeOH, 12h, rt, (23) = 97%, (III) a) drx DCM, NEt_3, 12h, rt, b) MeOH, NaOMe, (4) = 75%

3,5-Bis[2-(2-{2-[2-(adamantan-1-yloxy)ethoxy]ethoxy}ethoxy)ethoxy]benzoic acid pentafluorophenylester (20)

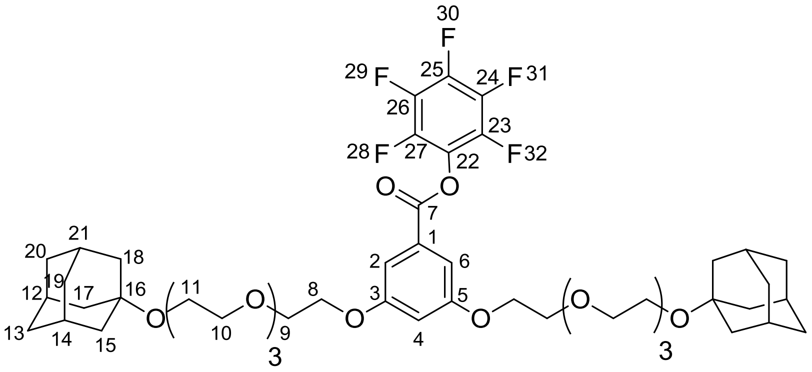

Compound **15** (150 mg, 0.14 mmol) and pentafluorophenol (25.2 mg, 0.15 mmol) were dissolved together in dry dichloromethane. The solution was cooled to 0 °C and *N,N'*-dicyclohexylcarbodiimide (31.3 mg, 0.152 mmol) was added. The temperature was allowed to equilibrate to room temperature and the mixture was stirred overnight. Afterwards solvent was removed under reduced pressure and column chromatography was applied to yield a yellow oil (142 mg, 0.11, 79%).

Molecular formula: $C_{49}H_{65}F_5O_{12}$.

^{1}H NMR (300 MHz, $CDCl_3$, 298 K): δ = 1.43-1.62 (m, 12H, 13-,19-,20-H), 1.61-1.77 (m, 12H, 15-,17-,18-H), 2.05 (s, 6H, 12-,14-,21-H), 3.19-3.72 (m, 24H, 10-11-H), 3.72-3.93 (m, 4H, 9-H), 3.96-4.19 (m, 4H, 8-H), 6.75 (t, *J* = 2.3 Hz, 1H, 4-H), 7.20-7.36 (m, 2H, 2-,6-H) ppm.

^{13}C NMR (75.5 MHz, $CDCl_3$, 298 K): δ = 30.61 (3 CH, 12-,14-,21-C), 36.56 (3 CH_2, 13-,19-,20-C), 41.58 (3 CH_2, 15-,17-,18-C), 59.36 (2 CH_2, 11-C), 68.04, 69.64, 70.72, 70.78, 71.00, 71.39 (14 CH_2, 8-10-C), 72.37 (2 C_q, 16-C), 108.52 (2 CH, 2-,6-C), 109.23 (CH, 4-C), 128.66 (C_q, 22-C), 130.09 (C_q, 1-C), 135.17 (C_q, 25-C), 139.66 (C_q, 23-,27-C), 140.38 (C_q, 24-,26-C), 160.21 (2 C_q, 3-,5-C), 183.88 (C_q, 7-C) ppm.

^{19}F-NMR (282 MHz, $CDCl_3$, 298 K): δ = -152.40 (29,-31-F), -157.94 (30-F), -162.30 (28-,31-F) ppm.

HRMS (*m/z*): calculated for $[C_{49}H_{65}F_5O_{12}Na]^+$: 963.4288, found: 963.4280

Dibenzyl {[(2-aminoethyl)azanediyl]bis(ethane-2,1-diyl)}dicarbamate (21)[4]

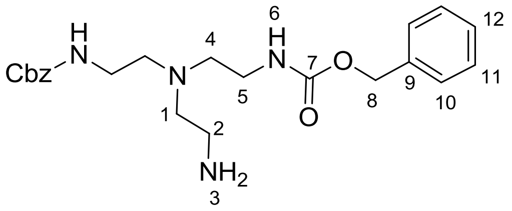

To a solution of tris(ethylamine) (0.489 g, 10 mmol) in absolute ethanol (20 mL) benzyl phenyl carbonate (1.52 g, 6.6 mmol) was added while being cooled with an ice bath. The mixture was stirred overnight at room temperature. The solvent was then removed under reduced pressure and water (50 mL) was added. The pH was adjusted to 3 by addition of aqueous HCl (2M). The solution was then extracted with dichloromethane. The pH of the aqueous phase was then adjusted to pH 10 and again extracted with dichloromethane. The solvent was evaporated. (1.4 g, 3.3 mmol, 66%)

Molecular formula: $C_{22}H_{30}N_4O_4$.

^{1}H NMR (300 MHz, $CDCl_3$, 298 K): δ = 7.59 – 7.01 (m, 8H, 10/11-H), 6.96 – 6.62 (m, 2H, 12-H), 5.75 (s, 2H, 6-H), 5.01 (s, 6H, 6/8-H), 3.18 (s, 10H, 2/3/4-H), 2.64 (t, *J* = 5.4 Hz, 4H, 5-H), 2.58 – 2.20 (m, 2H, 1-H).ppm.

^{13}C NMR (126 MHz, $CDCl_3$, 298 K): δ = 156.90 (1 C_q, 7-C) , 136.69 (1 C_q, 9-C), 129.50 (2 CH, 12-C), 128.46, 128.10, 119.62, 115.63, 128.02 (8 CH, 10/11-C), 66.59 (2 CH_2, 8-C), 56.05, 54.01 (3 CH_2, 3/4-C), 39.31, 39.08 (2 CH_2, 2/5-C) ppm.

HRMS (*m/z*): calculated for $[C_{22}H_{30}N_4O_4K]^+$: 415.2340, found: 415.2340.

Dibenzyl {[(2-{3,5-bis[2-(2-{2-[2-(adamantan-1-yloxy)ethoxy]ethoxy}ethoxy)ethoxy] benzamido}ethyl)azanediyl]bis(ethane-2,1-diyl)}dicarbamate (22)

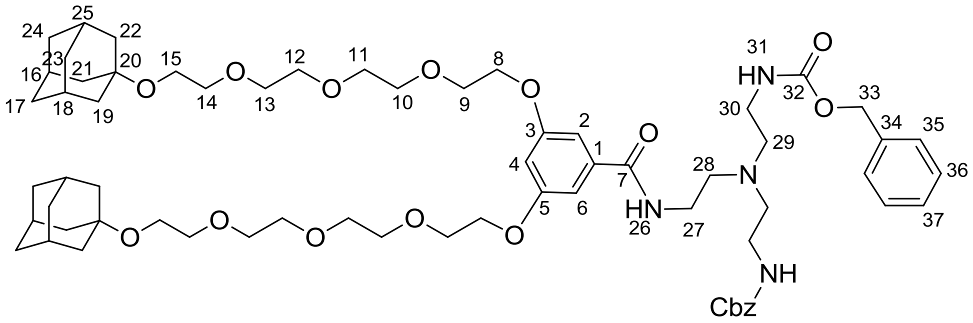

Compound **21** (185 mg, 0.44 mmol) was added to compound **20** (500 mg, 0.53 mmol) in dry dichloromethane at 0 °C. The solution was stirred overnight. Afterwards the solvent was evaporated and the crude product was purified by column chromatography (SiO_2: ethylacetate-methanol, (90:5), R_f: 0.45) (400 mg, 0.34 mmol, 77%).

Molecular formula: $C_{65}H_{94}N_4O_{15.}$

^{1}H NMR (300 MHz, $CDCl_3$) δ = 7.24 (s, 12H, 31/35-37-H), 7.01 (s, 2H, 2/6-H), 6.56 (t, *J* = 2.2 Hz, 1H, 4-H), 4.95 (s, 4H, 33-H), 4.32 (d, *J* = 7.9 Hz, 1H, 26-H), 4.05 (s, 4H, 8-H), 3.88 – 3.33 (m, 32H, 9-15/27-H), 3.24 (s, 4H, 30-H), 2.68 (d, *J* = 26.2 Hz, 6H, 28-/29-H), 2.12 (s, 6H, 16-,18-,25-H), 1.80 – 1.48 (m, 27H, 17-,19-,21-24-H) ppm.

^{13}C NMR (75 MHz, $CDCl_3$) δ = 167.66 (1 C_q, 7-C), 159.52 (2 C_q, 3-,5-C), 156.71 (1 C_q, 1-C), 136.28 (1 C_q, 34-C), 135.92 (2 CH, 37-C), 128.09, 127.62, 127.55 (8 CH, 35/36-C), 105.70 (2 CH, 2-,6-C), 104.62 (CH, 4-C), 72.06, 70.92, 70.40, 70.26, 70.23 (14 CH, 8-14-C), 69.25, 67.28 (2 C_q , 20-C), 66.28 (2 CH_2, 33-C), 58.91 (2 CH_2, 15-C), 48.90 (3 CH_2, 28/29-C), 41.12 (6 CH_2, 19-,21-,22-C), 38.60 (2 CH_2, 27/30-C), 36.11 (6 CH_2, 17-,23-,24-C), 30.17 (6 CH_2, 16-, 18- 25-C) ppm.

HRMS (*m/z*): calculated for $[C_{65}H_{94}N_4O_{15}Na]^+$: 1193.6608, found: 1193.6605.

3,5-Bis(2-(2-(2-(2-((3s,5s,7s)-adamantan-1-yloxy)ethoxy)ethoxy)ethoxy)ethoxy)-N-(2-(bis(2-aminoethyl)amino)ethyl)benzamide (23)

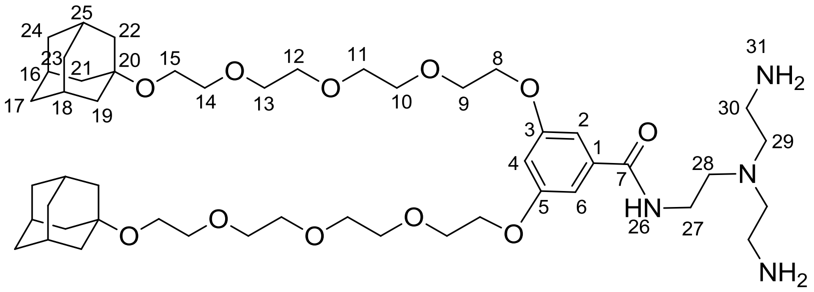

Compound **22** (400 mg, 0.34 mmol) was dissolved in dry MeOH. A catalytic amount of Pd/C was added and the mixture was stirred overnight under an H_2 atmosphere. (360 mg, 0.33 mmol, 97%).

Molecular formula: $C_{57}H_{88}N_4O_{13}$

^{1}H NMR (400 MHz, $CDCl_3$) δ = 8.52 (s, 1H, 26-H), 7.24 – 6.86 (m, 2H, 2-,6-H), 6.60 – 6.34 (m, 1H, 4-H), 4.27 (d, *J* = 7.9 Hz, 1H), 4.17 – 3.94 (m, 4H, 8-H), 3.88 – 3.74 (m, 4H, 9-H), 3.75 – 3.31 (m, 28H, 10-15-,27-H), 2.98 – 2.49 (m, 10H, 28-30-H), 2.41 (d, *J* = 20.4 Hz, 1H), 2.10 (s, 6H, 16-,18-,25-H), 1.95 – 1.80 (m, 1H), 1.77 – 1.44 (m, 27H, 17-,19,-21-24-H), 1.39 – 0.98 (m, 4H, 31-H) ppm.

^{13}C NMR (101 MHz, $CDCl_3$) δ 167.30 (1 C_q, 7-C), 159.79 (2 C_q, 3-,5-C), 157.08(1 C_q, 1-C), 106.29 (2 CH, 2-,6-C), 104.75 (CH, 4-C), 72.48, 71.46, 70.88, 70.78, 70.71, 70.68 (14 CH, 8-14-C), 69.88, 67.66 (2 C_q , 20-C), 59.42 (2 CH_2, 15-C), 49.23 (3 CH_2, 28/29-C), 41.66 (6 CH_2, 19-,21-,22-C), 38.83 (2 CH_2, 27/30-C), 36.63 (6 CH_2, 17-,23-,24-C), 30.68 (6 CH_2, 16-, 18- 25-C) ppm.

HRMS (*m/z*): calculated for $[C_{49}H_{82}N_4O_{11}Na]^+$: 925.5872, found: 925.5893.

N,N'-{[(2-{3,5-Bis[2-(2-{2-[2-(adamantan-1-yloxy)ethoxy]ethoxy}ethoxy)ethoxy] benzamido}ethyl)azanediyl]bis(ethane-2,1-diyl)}bis[4-(α-D-mannopyranosyloxy) butanamide] (4)

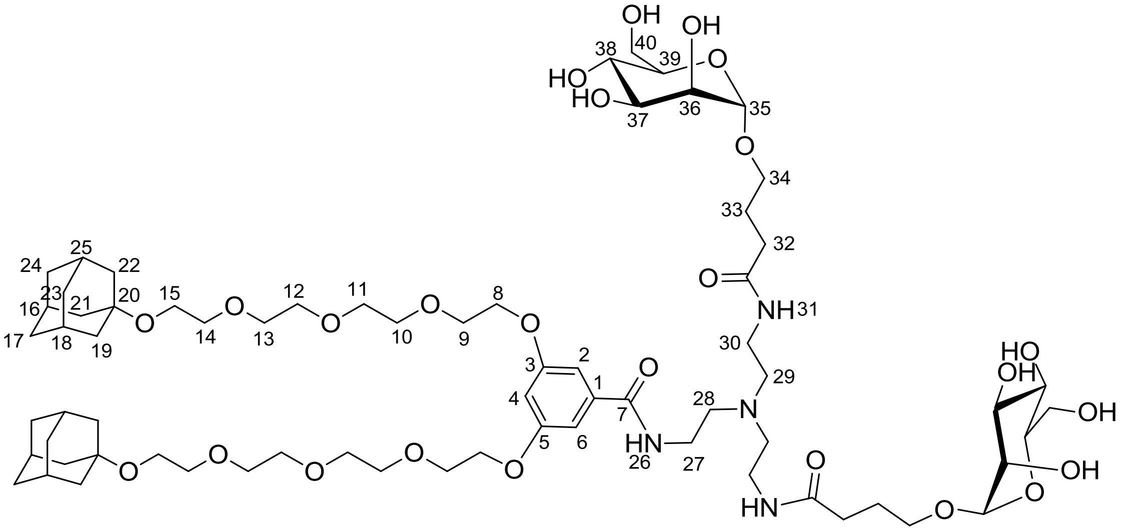

To a solution of compound **23** (150 mg, 0.17 mmol) in dry dichloromethane were added 2,3,4,6-tetra-O-acetyl-α-D-mannopyranoyl-oxybutanoic acid pentafluorophenol ester[5] (249 mg, 0.42 mmol) and triethylamine (50 mg, 69 µL, 0.50 mmol) at a temperature of 0 °C. Afterwards the temperature was equilibrated to room temperature and the solution was stirred for 5 h. The solvents were evaporated and the product was stirred overnight in MeOH. Then the solvents were again evaporated and the crude product was purified by column chromatography (SiO_2: ethylacetate-cyclohexane, (1:1) to ethylacetate-cyclohexane-methanol (1:1:0.1), R_f: 0.86) (115 mg, 0.06 mmol, 35%).

Molecular formula: $C_{69}H_{114}N_4O_{25}$.

^{1}H NMR (600 MHz, MeOD) δ = 7.07 (d, *J* = 2.2 Hz, 2H, 2/6-H), 6.69 (s, 1H, 4-H), 4.76-4.77 (d, *J* = 1.5 Hz, 2H, 35-H), 4.20 – 4.21 (m, 4H, 36-H), 3.82 – 3.90 (m, 6H, 8/37-H), 3.29 – 3.82 (m, 44H, 9-15-, 27/30/34-, 38-,39-, 40-H), 2.67-2.75 (m, 6H, 28/29-H), 2.25 – 2.29 (m, 4H, 32-H), 2.08 (s, 6H, 16-,18-,25-H), 1.86 – 1.92 (m, 12H, 19-, 21-, 22-H), 1.63 - 1.72 (dd, *J* = 34.3, 12.0 Hz, 12H, 17-,23-,24-H), 1.31 (s, 4H, 33-H) ppm.

^{13}C NMR (151 MHz, cd_3od) δ 180.22 (1 C_q, 7-C), 175.56 (2 C_q, 31-C), 169.54 (CH, 36-C) 161.39 (2 C_q, 3-,5-C), 137.54 (1 C_q, 1-C), 107.06 (2 CH, 2-,6-C), 105.80 (CH, 4-C), 101.39, 101.35 (2 CH, 35-C), 74.45 (1 CH_2, 40-C), 74.28, 73.57, 72.43, 72.09, 72.05, 71.97, 71.58, 71.39, 71.36, 71.32, 70.61 (14 CH, 8-14-C), 68.88, 68.51, 68.47, 68.27 (4 CH, 34/38/39-C), 67.68 (2 C_q , 20-C), 62.66, 62.56 (2 CH, 37-C), 60.25 (2 CH_2, 15-C), 54.51 (3 CH_2, 28/29-C), 42.38 (6 CH_2, 19-,21-,22-C), 39.15, 38.56 (2 CH_2, 27/30-C), 37.34 (6 CH_2, 17-,23-,24-C), 35.55, 33.72 (2 CH_2, 32-C), 31.83 (6 CH_2, 16-, 18- 25-C), 27.48, 26.65, (2 CH_2, 33-C).

HRMS (*m/z*): calculated for $[C_{69}H_{114}N_4O_{25}Na]^+$: 1421.7664, found: 1421.7684

1. Ravoo, B. J.; Darcy, R., *Angew. Chem. Int. Ed.* **2000**, *39*, 4324-4326.

2 . Mulder, A.; Onclin, S.; Péter, M.; Hoogenboom, J.P.; Beijleveld, H.; ter Maat, J.; García-Parajó, M.F.; Ravoo, B.J.; Huskens, J.; van Hulst, N.F.; Reinhoudt, D.N., *Small* **2005**, 1, 242-253.

3. Baars, M. W. P. L; Kleppinger, R.; Koch, M. H. J.; Yeu, S.; Meijer, E. W., *Angew. Chem. Int. Ed.* **2000**, *39*, 1285-1288.

4. Moore, E. G., Xu, J., Jocher, C. J., Corneillie, T. M., Raymond, K. N., *Inorg. Chem.*, **2010,** *49*, 9928-9939.

5. Wendeln, C.; Rinnen, S.; Schulz, C.; Kaufmann, T.; Arlinghaus, H. F.; Ravoo, B. J., *Chem. Eur. J.* **2012,** *18*, 5880–5888.
